# Structural Basis for Redox Regulation of Cytoplasmic and Chloroplastic Triosephosphate Isomerases from *Arabidopsis thaliana*

**DOI:** 10.3389/fpls.2016.01817

**Published:** 2016-12-06

**Authors:** Laura M. López-Castillo, Pedro Jiménez-Sandoval, Noe Baruch-Torres, Carlos H. Trasviña-Arenas, Corina Díaz-Quezada, Samuel Lara-González, Robert Winkler, Luis G. Brieba

**Affiliations:** ^1^Laboratorio Nacional de Genómica para la Biodiversidad, Centro de Investigación y de Estudios Avanzados del Instituto Politécnico NacionalIrapuato Guanajuato, Mexico; ^2^Departamento de Biotecnología y Bioquímica, CINVESTAV Unidad IrapuatoIrapuato Guanajuato, Mexico; ^3^División de Biología Molecular, Instituto Potosino de Investigación Científica y Tecnológica A.C.San Luis Potosí, Mexico

**Keywords:** triosephosphate isomerase, *Arabidopsis thaliana*, X-ray structure, thiol-based redox regulation, glutathionylation

## Abstract

In plants triosephosphate isomerase (TPI) interconverts glyceraldehyde 3-phosphate (G3P) and dihydroxyacetone phosphate (DHAP) during glycolysis, gluconeogenesis, and the Calvin-Benson cycle. The nuclear genome of land plants encodes two *tpi* genes, one gene product is located in the cytoplasm and the other is imported into the chloroplast. Herein we report the crystal structures of the TPIs from the vascular plant *Arabidopsis thaliana* (AtTPIs) and address their enzymatic modulation by redox agents. Cytoplasmic TPI (cTPI) and chloroplast TPI (pdTPI) share more than 60% amino acid identity and assemble as (β-α)_8_ dimers with high structural homology. cTPI and pdTPI harbor two and one accessible thiol groups per monomer respectively. cTPI and pdTPI present a cysteine at an equivalent structural position (C13 and C15 respectively) and cTPI also contains a specific solvent accessible cysteine at residue 218 (cTPI-C218). Site directed mutagenesis of residues pdTPI-C15, cTPI-C13, and cTPI-C218 to serine substantially decreases enzymatic activity, indicating that the structural integrity of these cysteines is necessary for catalysis. AtTPIs exhibit differential responses to oxidative agents, cTPI is susceptible to oxidative agents such as diamide and H_2_O_2_, whereas pdTPI is resistant to inhibition. Incubation of AtTPIs with the sulfhydryl conjugating reagents methylmethane thiosulfonate (MMTS) and glutathione inhibits enzymatic activity. However, the concentration necessary to inhibit pdTPI is at least two orders of magnitude higher than the concentration needed to inhibit cTPI. Western-blot analysis indicates that residues cTPI-C13, cTPI-C218, and pdTPI-C15 conjugate with glutathione. In summary, our data indicate that AtTPIs could be redox regulated by the derivatization of specific AtTPI cysteines (cTPI-C13 and pdTPI-C15 and cTPI-C218). Since AtTPIs have evolved by gene duplication, the higher resistance of pdTPI to redox agents may be an adaptive consequence to the redox environment in the chloroplast.

**Graphical Abstract:**
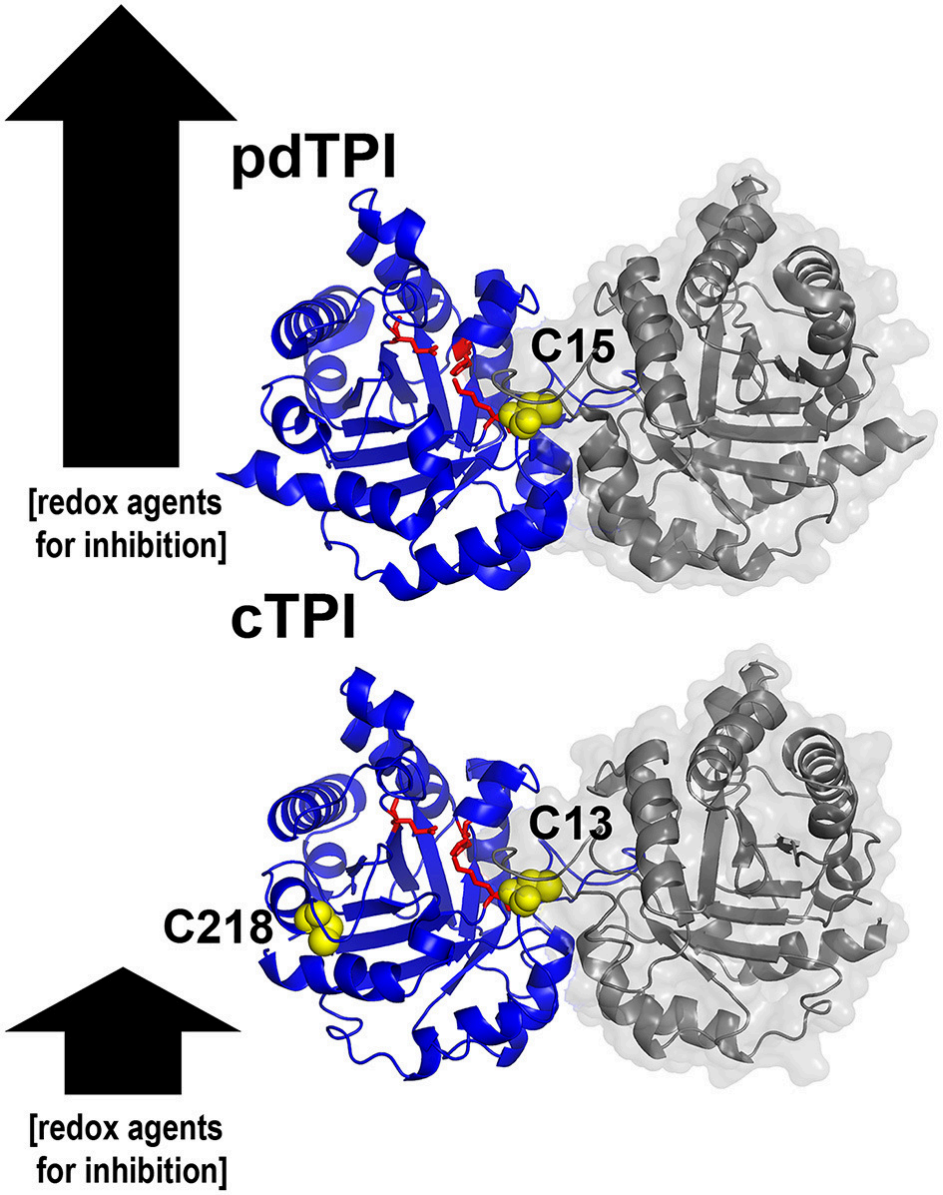
**Structural basis for the differences in redox regulation between triosephosphate isomerase from ***Arabidopsis thaliana*****. Ribbon representation of cytoplasmic and chloroplastic triosephosphate isomerases showing their accesible cysteines. The arrows illustrate the relative amount of redox agents necessary for enzymatic inhibition.

## Introduction

Triosephosphate isomerase (TPI) interconverts glyceraldehyde 3-phosphate (G3P) and dihydroxyacetone phosphate (DHAP). The structure of this enzyme consists of eight alternate β-strands and α-helices, dubbed TIM or (β/α)_8_ barrel fold (Banner et al., 1975). Crystal structures of more than 40 TPIs from different species are deposited in the Protein Data Bank (PDB). Surprisingly, the only crystal structure of a TPI from a photosynthetic organism is that of the chloroplast TPI from *Chlamydomonas reinhardtii* (CrTPI) (Zaffagnini et al., 2014). One or various cytoplasmic and chloroplast TPIs (cTPI and pdTPI), are present in plant genomes. cTPIs are involved in glycolysis, whereas chloroplast enzymes participate in the Calvin-Benson cycle (Turner et al., 1965; Kurzok and Feierabend, 1984; Tang et al., 2000; Chen and Thelen, 2010). In contrast, in unicellular green algae, the first reactions of the glycolytic pathway from glucose phosphorylation to triosephosphate isomerization occur inside the chloroplast (reviewed in Johnson and Alric, 2013) and unicellular green algae only contain one TPI isoform.

Plant TPIs are subject to transcriptional regulation and are involved in developmental processes. For example, in rice the accumulation of toxic methylglyoxal (MG) leads to an increase in cTPI transcription and enzymatic activity (Sharma et al., 2012). In *A. thaliana* the lack of pdTPI results in plants unable to transit into the reproductive stage or suffer stunted growth and abnormal chloroplast development. These physiological abnormalities are attributed to the accumulation of DHAP and MG (Chen and Thelen, 2010). In the leaves of *Solanum chacoense*, cTPI activity increases during growth and decreases when growth slows down (Dorion et al., 2005). Changes in TPI activity are linked to the anaplerotic role of glycolysis, in which pyruvate is synthesized to replenish acetyl-CoA for the citric acid cycle. It has been suggested that this process has a regulatory effect on the carbon requirements for plant development (Dorion et al., 2005).

In plants, several enzymes are regulated under stress conditions by redox-based signaling mechanisms, such as glutathionylation and nitrosylation (Dixon et al., 2005; Shapiro, 2005; Poole and Nelson, 2008; Dalle-Donne et al., 2009; Gao et al., 2009; Zaffagnini et al., 2012a,b). Proteomic strategies identified cTPIs as targets of glutathionylation and nitrosylation (Ito et al., 2003; Dixon et al., 2005) but failed to identify pdTPIs. The effects of glutathionylation are diverse among TPIs. For example, in cTPI from *A. thaliana* the addition of GSSG causes a total loss of enzymatic activity and this loss in activity is reversed by the addition of GSH (Ito et al., 2003), whereas the addition of GSSG only produces a slight reduction in the activity of *C. reinhardtii* pdTPI (Zaffagnini et al., 2014) and no effect on yeast TPI (Shenton and Grant, 2003).

Structure-function studies of TPIs from animals, protozoa, and bacteria illustrate that these enzymes are regulated by diverse mechanisms such as: altering dimer-monomer equilibrium, deamination, phosphorylation, binding to competitive inhibitors or binding to thiol-conjugated reagents (Ralser et al., 2006; Olivares-Illana et al., 2007; Lee et al., 2010; Enríquez-Flores et al., 2011; Grüning et al., 2014; Lara-Gonzalez et al., 2014, 2015; de la Mora-de la Mora et al., 2015). In contrast, no structure-function studies of TPIs from land plants have been carried out to date. The determination of the crystal structure of CrTPI as the only structure-function study of a TPI from a photosynthetic organism (Zaffagnini et al., 2014) is a breakthrough, however the genome of *C. reinhardtii* does not contain a cTPI gene, making it impossible to establish a direct comparison between TPI isoforms. Furthermore, phylogenetic analysis indicates that plant pdTPIs have a eukaryotic origin, derived from gene duplication of cTPI and the addition of the chloroplast targeting sequence (Schmidt et al., 1995; Reyes-Prieto and Bhattacharya, 2007). Proteomic analyses show that cTPIs but not pdTPIs are targets of glutathionylation and nitrosylation (Ito et al., 2003; Dixon et al., 2005), suggesting that although pdTPIs and cTPIs share the same evolutionary origin, they have evolved to carry out different responses to redox agents in relation to their cellular localization. Thus, the aim of this study was to investigate at a structural level the mechanisms that account for the possible selective enzymatic redox modulation in pdTPIs and cTPIs.

## Materials and methods

### Plasmid construction for expression of AtTPIs and site-directed mutagenesis

cTPI was amplified with primers: cTPI-N-term, 5′-GACGACGAGAATTCATGGCCAGAAAGTTCTTCGTCG-3′ and cTPI-C-term, 5′-GACGACGACTCGAGTTAGGCACTTTTCTTCACCTCTGC-3′ from cDNA; pdTPI was amplified from the pET200/D-TOPO vector with primers: pdTPI-N-term, 5′-GACGACGAGAATTCATGGCTGGATCCGGAAAGTTTTTC-3′ and pdTPI-C-term, 5′-GACGACGACTCGAGTCAAGCAGCAACTTTCTTCGACG-3′ and subcloned into the EcoRI and XhoI restriction sites of a pET21a vector and a modified pET28b vector in which the enterokinase site was substituted for a precision protease recognition site. Site-directed mutagenesis was carried out using the Quick-Change Mutagenesis Kit.

### Protein expression and purification

AtTPIs were expressed in BL21 *E. coli* Arctic (*DE3*) or BL21 *E. coli* (*DE3*) Δ*tpi* cells for crystallographic and biochemical studies respectively. The bacterial pellet was recovered and resuspended in 25 mM Tris-HCl pH 8.0, 150 mM NaCl and purified by metal chelate affinity chromatography (IMAC), using a His-Trap FF column (GE Healthcare, USA). Protein was dialyzed in 25 mM Tris-HCl pH 8.0, 150 mM NaCl. After the affinity purification the protein was concentrated using centrifugal ultrafiltration units of 10 kDa MWCO (Millipore, USA), and then purified by size-exclusion chromatography using an ÄKTA FPLC UPC-900 System (Amersham Biosciences), loading the samples on a Superdex 75 Column (GE Healthcare, USA) and concentrating the fractions containing the target protein to 172 μM before storing at 4°C.

### Protein reduction

Concentrated AtTPIs were reduced with 20 mM dithiothreitol (DTT) for 1 h at room temperature. Proteins were desalted using a prepacked Sephadex G-25 column equilibrated with 25 mM Tris-HCl pH 8.0, 150 mM NaCl.

### *In vivo* functional assays and determination of catalytic parameters of AtTPIs

*In vivo* complementation assays, and catalytic activity of AtTPIs were measured as previously reported (Rozacky et al., 1971; Sullivan et al., 2011). Catalytic activity of AtTPIs was measured spectrophotometrically by a coupled enzyme assay with α-GDH in the direction G3P to DHAP(Rozacky et al., 1971). Assays were performed according to Benítez-Cardoza et al. (2001), by adding 100 μL of 0.1 TEA pH 7.4, containing 10 mM EDTA, 0.01% BSA, 0.2 mM NADH (SIGMA, USA), 1 μg α-GDH (Roche, Germany) and 1 mM of D-L glyceraldehyde 3-phosphate (SIGMA, USA). The reaction was started by addition of AtTPIs and activity was calculated by the decrease in absorbance at 340 nm, monitored each 30 s using an Infinite M1000 reader (TECAN, Switzerland). For the determination of the apparent kinetic constants (K_m_ and k_cat_), assays were carried out by increasing G3P concentrations, from 0 to 4.0 mM.

### Determination of free sulfhydryl groups

The free sulfhydryl concentration of AtTPIs was determined spectrophotometrically with DTNB (Ellman, 1959). For this assay 90 μL of previously quantified protein was added to a solution containing 200 μM of DTNB (SIGMA, USA) in 100 mM sodium phosphate pH 8.0. The absorbance at 412 nm was determined after 30 min. of incubation at room temperature. A molar extinction coefficient of 14,150 M^−1^cm^−1^ was used to calculate the number of titrated sulfhydryl groups.

### Redox treatments of AtTPIs

Oxido-reduction treatments were performed by incubating 1 μM of reduced AtTPIs in 100 mM TEA, pH 7.4, containing 10 mM EDTA with 20 mM DTT, 0.2 mM H_2_O_2_, 2.5 mM GSSG or 1 mM diamide for 2 h at 37°C. Untreated proteins under the same incubation conditions were used as controls and their activity was taken as reference (100% of TPI activity).

### Western blot for glutathionylation of AtTPIs

Wild-type and mutant AtTPIs were diluted to 3.45 μM and incubated in a buffer containing 100 mM TEA pH 7.4, 10 mM EDTA and 10 mM GSSG overnight at 4°C. After incubation, protein samples were separated by non-reducing SDS-PAGE and analyzed by Western blotting, using a primary mouse anti-Glutathione monoclonal antibody (1:250 dilution, VIROGEN, USA), and an anti-mouse secondary antibody coupled to peroxidase (dilution 1:5000, Thermo Scientific). Signals were visualized by enhanced chemiluminescence, using CL-Xposure Film (Thermo Scientific, USA).

### Effect of methyl methanethiosulfonate (MMTS) on AtTPI activity

Reduced AtTPIs were diluted to 0.184 nM (10 ng/ml) in 100 mM TEA pH 7.4, 10 mM EDTA. Protein samples were incubated with increasing concentrations of MMTS (0–400 μM) for 3 h at room temperature. Aliquots were withdrawn and activity was measured at 340 nm.

### Effect of GSSG on AtTPI activity

Reduced AtTPIs were diluted to 0.184 μM in 100 mM TEA pH 7.4, 10 mM EDTA with increasing concentrations of GSSG (0–25 mM). Reactions were incubated during 3 h at room temperature. After derivatization, proteins were diluted to reach a final concentration of 0.184 nM (10 ng/ml) and activity was measured at 340 nm.

### Mass determination of glutathionylated proteins by electrospray ionization mass spectrometry

cTPI and pdTPI were diluted to 34.4 μM and incubated with 0.25, 2.5, and 25 mM of GSSG overnight. Subsequently, proteins were extensively dialyzed against 50 mM Tris-HCl pH 8.0, for GSSG removal. Protein samples were dialyzed twice against water, mixed with methanol (2:1 v/v) and acidified with formic acid (2.8% final concentration). Electrospray ionization spectra were collected on a LTQ Velos ion trap mass spectrometer and converted to mzML file format using ProteoWizard (Kessner et al., 2008). The sum spectra were visualized in TOPPView (Sturm et al., 2008), a Gaussian noise filter (5 Da width) was applied to smooth the signals. MASSyPup64 and ESIprot were used as data processing platforms (Winkler, 2010, 2015).

### Effects of mutagenesis and glutathionylation on the oligomerization of AtTPIs

For the determination or the oligomeric state of AtTPIs (wild-type and mutants), analytical size-exclusion chromatography was performed on a Superdex 75 10/300GL column (GE Healthcare) connected to an ÄKTA FPLC System (Amersham Biosciences). The column was calibrated with standard proteins using the Bio-Rad gel filtration standard, following the manufacturers instructions. Proteins were eluted at a flow-rate of 0.5 mL/min with 100 mM TEA, pH 7.4 containing 10 mM EDTA and 1 mM DTT. To study the effect of glutathionylation, AtTPIs were incubated with 5 mM GSSG over night at room temperature before loading onto the column.

### Crystallization, data collection, and structure determination

cTPI and pdTPI were concentrated to 0.34 mM in a buffer containing 20 mM Tris pH 8, 50 mM NaCl and 2 mM DTT. A sparse matrix crystallization screen of 96 conditions was assayed at 21°C using the hanging drop method. cTPI crystals grew in 0.1 M Tris-HCl, pH 8.5, 0.2 M magnesium chloride hexahydrate, 30% PEG 400, and 0.16 M magnesium chloride hexahydrate, whereas pdTPI crystals grew in 0.1 M HEPES, pH 7.5, and 1.4 M sodium citrate tribasic dihydrate. Data were collected from single crystals using synchrotron radiation at Advanced Photon Source. Datasets were processed with MOSFLM (Battye et al., 2011) and scaled with AIMLESS, from the CCP4 Software Suite. The X-ray structures of both AtTPIs were solved by molecular replacement in the space group P2_1_ 2_1_ 2 for cTPI and P6_5_ 2 2 for pdTPI with the program PHASER (McCoy et al., 2007). The electrostatic potentials of the molecular surface were calculated with PBEQ-solver (Jo et al., 2008), which uses the Poisson–Boltzmann equations module from the biomolecular simulation program CHARMM (Brooks et al., 2009). Parameters for calculation of electrostatic potential surface were the default on the PBEQ Solver (PBEQ Physical Parameters). The resulting maps were then loaded in Pymol for visualization and image generation (Delano, 2002). PDB accession numbers, data collection and refinement statistics are presented in Table S1.

## Results

### Expression, purification, and sequence analysis of AtTPIs

**Figure 1:**
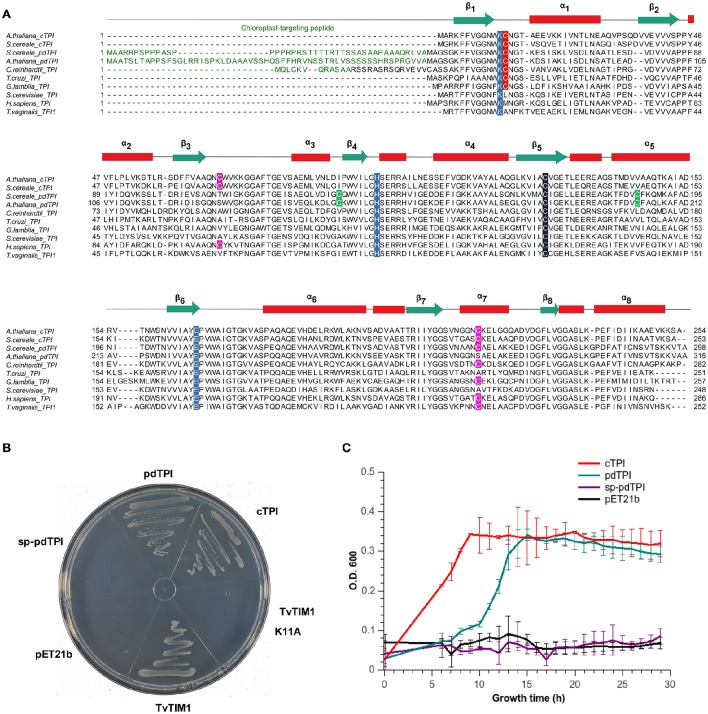
**AtTPIs are functional TPIs that harbor isoform specific cysteines**. **(A)** Multiple sequence amino acid alignment of selected TPIs showing their secondary structural elements. Chloroplast targeting sequences are colored in green. Catalytic residues (K12, H95, and E166 according to yeast TPI numeration) are highlighted in blue. Cysteine residues are highlighted as follows: the universally TPI conserved C127 in black; loop1 interface cysteine in red (C13 or C15), a cysteine conserved in plant cTPIs in purple, cysteines conserved in pdTPI isoforms are colored in green, and the α-helix 7 cysteine in pink. **(B)** LB agar plates showing the *in vivo* complementation of *E. coli* BL21 (DE3) ΔTPI cells by plasmids expressing cTPI, pdTPI, and TvTIM1. **(C)** Growth kinetics showing the complementation of BL21 (DE3) Δtpi cells by plasmids overexpressing AtTPIs after 30 h of growth.

*Arabidopsis thaliana* contains two nuclear-encoded AtTPI genes (Arabidopsis-Genome-Initiative, 2000; Chen and Thelen, 2010). At3g55440 encodes a cTPI, whereas At2g21170 encodes a pdTPI that is translocated into the chloroplast. The plastid gene encodes two protein isoforms. The At2g21170.1 (pdTPI) transcript encodes a canonical pdTPI, whereas the At2g21170.2 (sp-pdTPI) transcript expresses a splicing variant with a deletion of 9 amino acids (Chen and Thelen, 2010). The absence of the active site lysine (K14) in sp-pdTPI suggests that this isoform is inactive (Figure S1) (Alber et al., 1981; Schliebs et al., 1996). A multiple sequence alignment between TPIs from diverse organisms highlights particular features of the AtTPIs (Figure 1A and Figure S1). For example, the predicted N-terminal chloroplast targeting peptide of pdTPI is present in plastid TPIs from other species such as *C. reinhardtii* or *Secale cereale*. cTPI and the processed form of pdTPI (after the removal of the chloroplast transit peptide) share 62.3% of amino acid identity. cTPI and pdTPIs contain the catalytic triad (Lys, His, and Glu) and archetypical secondary structural elements such as loop 3, the phosphate binding loop, loop 6, and the YGGS motif. Also, each AtTPI harbors four cysteines in their primary structure (Figure S1).

### cTPI and pdTPI enzymes are functional *in vivo*

As *E. coli* strains devoid of a functional TPI gene are unable to grow on minimal medium due to the accumulation of methylglyoxal (MG) (Saab-Rincón et al., 2001; Gnerer et al., 2006), the functionality of AtTPIs was determined by complementation assays (Sullivan et al., 2011). The appearance of colonies where bacteria were transformed with plasmids carrying recombinant cTPI and pdTPI and a plasmid control expressing a TPI from *T. vaginalis* (TvTIM1) (Figueroa-Angulo et al., 2012) indicates that cTPI and pdTPI are catalytically active (Figure 1B). sp-pdTPI and the catalytically incompetent TvTIM1-K11A mutant were unable to achieve complementation indicating that sp-pdTPI is not catalytically active. Bacterial growth curves in liquid LB media showed an exponential phase and a stationary phase in bacterial cultures harboring cTPI and pdTPI plasmids, as expected no growth was observed in cultures containing sp-pdTPI or the vector alone (Figure 1C). Recombinant cTPI and pdTPI were expressed in bacteria and purified to homogeneity using immobilized-metal affinity chromatography (IMAC) and gel filtration, however we were unable to purify sp-pdTPI to homogeneity (Figure S2). The failure to obtain a heterologous sp-pdTPI protein correlates with the fact that this variant lacks the first β-strand that participates in folding of the inner TIM barrel. For enzymatic studies we used an *E. coli* strain devoid of TPI activity to avoid potential presence of endogenous co-purified TPI.

### AtTPIs are catalytic homodimers

Purified recombinant AtTPIs have a molecular mass of approximately 29 kDa. Their oligomeric state profile showed that these proteins were retained in a gel filtration column with a Stokes radius that corresponds to a molecular mass of 54 kDa indicating that AtTPIs assemble as dimers (Figure S2). cTPI was characterized by Shih and coworkers (Shih, 1994), but no information on its kinetic constants is available, whereas for pdTPI a V_max_ of 4.69 mM min^−1^ μg^−1^ was previously determined (Chen and Thelen, 2010). Our steady-state kinetic characterization indicates that cTPI catalyzes the G3P to DHAP isomerization with a K_m_ of 0.48 mM and a turnover number (k_cat_) of 2565 s^−1^, corresponding to a catalytic efficiency of 5.34 × 10^6^ M^−1^ s^−1^; whereas pdTPI displayed a K_m_ of 0.40 mM and a k_cat_ of 2337 s^−1^, corresponding to a catalytic efficiency of 5.48 × 10^6^ M^−1^ s^−1^. The observed K_m_ values are comparable to constants from other plant TPIs, such as pea seed TPI, with a value of 0.44 mM (Turner et al., 1965), rye cTPI with a value of 0.6 mM, and its chloroplast counterpart with a value of 0.68 mM (Kurzok and Feierabend, 1984), spinach pdTPI (Tang et al., 1999) and TPIs from other organisms such as *P. falciparum* (Samanta et al., 2011) *S. cerevisiae* (Gonzalez-Mondragon et al., 2004), and *T. cruzi* (Reyes-Vivas et al., 2001; Table 1). In our hands pdTPI presents a k_cat_ of 2337 s^−1^ that contrasts with the value of 424 s^−1^ previously reported (Chen and Thelen, 2010).

**Table 1:** **Catalytic parameters of plant TPIs**.

**Enzyme**	**Km (mM)**	**kcat (s^−1^)**	**kcat/Km (M^−1^ s^−1^)**	**References**
cTPI	0.48±0.05	2565±43.9	5.34 × 10^6^	This work
pdTPI	0.40±0.02	2337±41.6	5.84 × 10^6^	This work
pdTPI	N/R	424	N/R	Chen and Thelen, 2010
Spinach TPI	0.68	4510	6.63 × 10^6^	Tang et al., 1999
Pea seed TPI	0.44	N/R	N/R	Turner et al., 1965
*C. reinhardtii* TPI	3.04±0.1	3372±37	0.112 × 10^6^	Zaffagnini et al., 2014
*G. lamblia* TPI	0.78±0.1	7666±267	9.83 × 10^6^	Enríquez-Flores et al., 2011
*P. falciparum* TPI	0.35±0.1	4300±300	1.23 × 10^7^	Samanta et al., 2011
*S. cerevisiae* TPI	1.1±0.4	4700±700	4.27 × 10^6^	Gonzalez-Mondragon et al., 2004
*T. cruzi* TPI	0.48±0.01	4333.33	9.03 × 10^6^	Reyes-Vivas et al., 2001

*N/R, Not reported*.

### Crystal structures of AtTPIs

**Figure 2:**
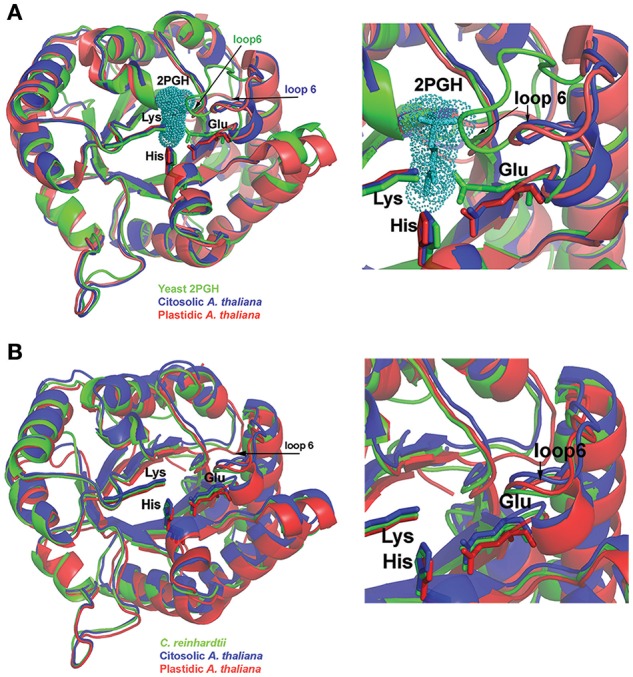
**Structural comparison between AtTPIs and other TPIs. (A)** Structural comparison between AtTPIs and yeast TPI in complex with PGH. PGH is depicted in a van der Waals representation and the catalytic residues Lys, His, and Glu are represented in a ball-stick representation. A zoom of the superimposition (right) denotes that AtTPIs crystallized in an open conformation in which loop 6 is moved out of the active site. **(B)** Superimposition of cTPI, pdTPI, and CrTPI. The catalytic residues Lys, His, and Glu are in a stick representation. A closer look at the active site (right) illustrates the positions of loop 6 and catalytic Glu between open and closed conformations.

**Figure 3:**
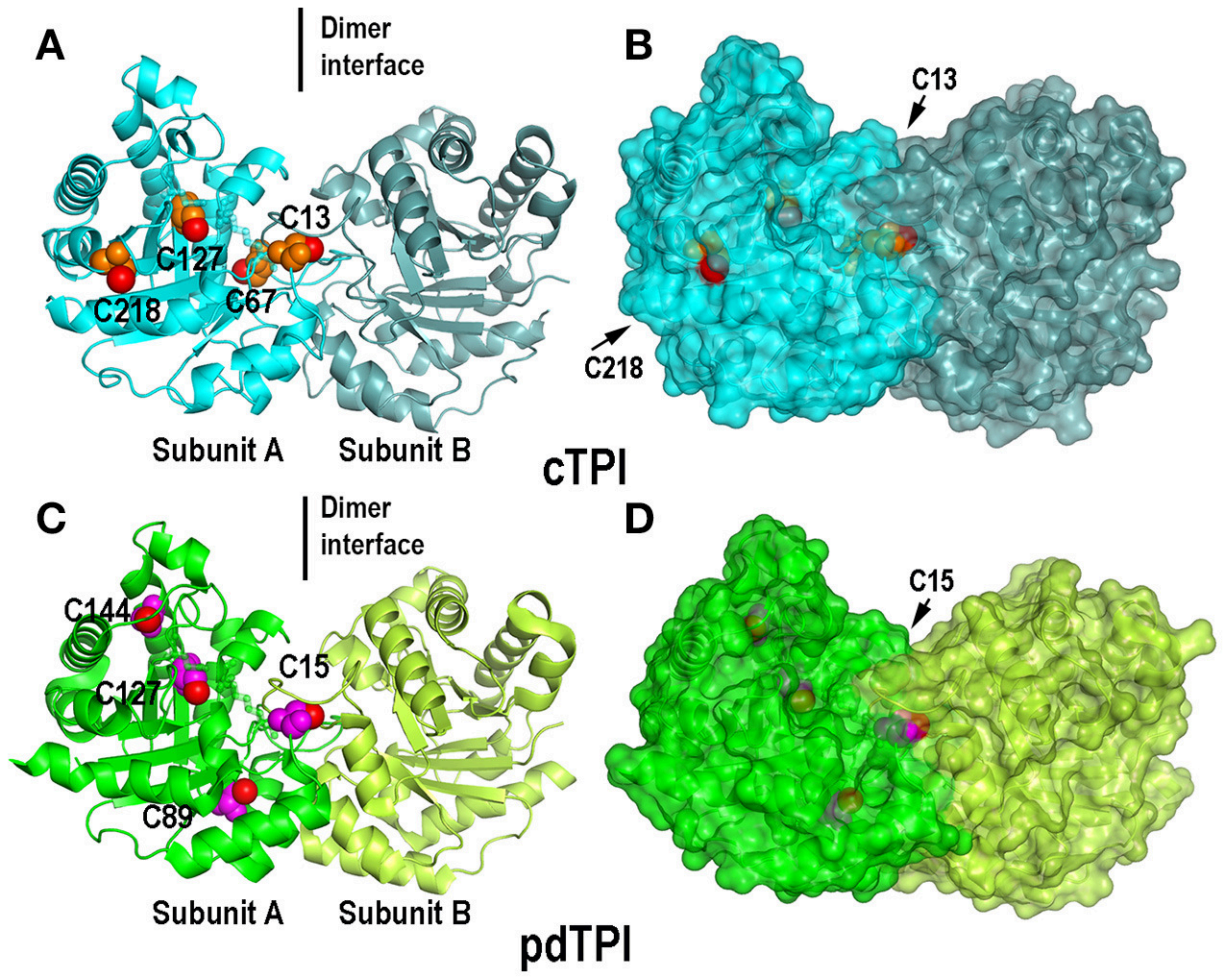
**Crystal structures of dimeric AtTPIs showing their isoform specific cysteines. (A)** Ribbon representation of dimeric cTPI. Subunit A is colored in cyan and subunit B is colored in steel blue. The isoform specific cysteines cTPI-C13, cTPI-C67, cTPI-127, and cTPI-C218 are represented as spheres, the thiol group is colored in red and the rest of the amino acid in orange. **(B)** Surface representation of cTPI. The thiol group of residues cTPI-C13 and cTPI-C218 are exposed to the solvent, whereas cTPI-C67 and cTPI-127 are buried in the hydrophobic core. **(C)** Ribbon representation of dimeric pdTPI. Subunit A is colored green and subunit B is colored lime. The isoform specific cysteines pdTPI-C15, pdTPI-C89, pdTPI-127, and pdTPI-C144 are represented as spheres, the thiol group is colored red and the rest of the amino acid in magenta. **(D)** Surface representation of pdTPI. The thiol group of residue cTPI-C15 is exposed to the solvent, whereas the rest of the thiol groups are buried in the hydrophobic core. Although residue pdTPI-c89 is solvent exposed, the sulfhydryl group of pdTPI-c89 points toward the hydrophobic core.

Crystal structures of cTPI and pdTPI were solved at 1.7 and 2.15 Å respectively (Table S1). The recombinant construct that encodes pdTPI eliminates the first 59 amino acids that correspond to the predicted chloroplast targeting sequence. In this work the numeration for the initial pdTPI amino acid starts after the predicted chloroplast targeting sequence. Clear electron density was observed for all residues except the first four amino acids of pdTPI and the initial methionine and last two amino acids of cTPI. The three-dimensional structures of AtTPIs confirm that both enzymes assembled as dimers with asymmetric units containing two monomers that fold into a canonical (β-α)_8_ or TIM-barrel. In a TIM barrel, eight antiparallel β-strands form the inner part of the barrel and eight α-helices form the solvent exposed part of the barrel (Figures 2A, 3). The Cα of cTPI and pdTPI superimpose with a root mean square deviation (rmsd) of 0.4 Å and are practically superimposable with the crystal structure of CrTPI (Zaffagnini et al., 2014; Figure 2B). A closer look at the superimposition shows that the position of the catalytic amino acids (K12, H95, and E165, using the numeration of yeast TPI), the phosphate binding loop, and loop 6 are practically identical (Figure 2). Crystal structures of several TPIs in the presence and absence of ligands showed that TPIs present two main loop 6 conformations. In the absence of ligand, TPIs present an open conformation whereas in the presence of ligand a closed conformation is observed (Wierenga et al., 1992). Crystal structures of TPIs with the transition state analog phosphoglycolohydroxamate (PGH) have shown the movement of loop 6 toward the active site. A structural superimposition between AtTPIs and yeast TPI in complex with PGH illustrates that AtTPIs crystallized in an open conformation. This is in contrast to the crystal structure of yeast TPI in which loop 6 moves to assemble the active site and the catalytic residue E165 is reoriented (Figure 2A). The dimeric interface in TPIs is held by a swapping interaction of loop 3 between subunits and by a large hydrophobic region (Figure 3 and Figure S3). The interface area for the cTPI dimer is 1478 Å^2^ and the interface for the pdTPI dimer is 1597 Å^2^. These areas are in agreement with the observed values of several previously described TPIs (Lolis et al., 1990; Mande et al., 1994; Delboni et al., 1995; Velanker et al., 1997; Lara-Gonzalez et al., 2014).

### Surface accessible cysteines in AtTPIs

Since the crystal structures of TPIs are similar between species we focused on the position of cysteine residues that can function as regulatory elements. A structural superimposition between cTPI and pdTPI depicts the localization of isoform-specific cysteines (Figure 3, and Figure S4). A surface projection of both structures shows that residues C13 and C218 of cTPI and residues C15 and C89 of pdTPI are solvent exposed (Figure 3 and Figure S4). However, their structural context is different: residues cTPI-C13 and pdTPI-C15 form part of the dimer interface, pdTPI-C89 is located at the N-terminus of α-helix 3 and cTPI-C218 at the C-terminus of α-helix 7. Considering that the possibility of a cysteine residue to react with GSSG is correlated with its accessible surface area (ASA), we analyzed the possible role of the exposed AtTPIs cysteines as glutathione targets. Residue cTPI-C13 presents an ASA of 2.6 and 2.8 Å^2^ for monomers A and B respectively, whereas residue C218 presents an ASA of 6.7 and 2.6 Å^2^ for monomers A and B respectively. In pdTPI, residue C15 presents an ASA of 2.8 and 3.3 Å^2^, whereas residue C89 presents an ASA of 8.2 and 8.5 Å^2^ (Table 2). The sulfhydryl groups (-SH) of pdTPI-C15, cTPI-C13 and cTPI-C218 are solvent exposed, whereas the -SH group of pdTPI-C89 points toward the hydrophobic core (Figure S4). Residues cTPI-C13 and pdTPI-C15 are located in loop 1 at an equivalent structural position, whereas residue cTPI-C218 is located in α–helix 7 (Figure S4). Thus, ASA calculations indicate that cTPI and pdTPI contain 2 and 1 accessible cysteines per monomer. This observation is validated by the quantification of free thiols by DTNB indicating that cTPI and pdTPI contain 2 and 1.6 accessible thiols per monomer, respectively (Table 3). As cTPI-C13 and pdTPI-C15 are part of the dimer interface, we measured their ASAs as monomers, using the coordinates from monomer A and suppressing dimer formation. Surface accessibility calculation using structural data from monomeric AtTPIs indicate that the ASA of cTPI-C13 increases from 2.68 to 129.3 Å^2^ and the ASA of pdTPI-C15 increases from 2.8 to 127.2 Å^2^ (Table 2).

**Table 2:** **Theoretical accessible surface area of cysteine residues of AtTPIs**.

**Residue**	**Dimer ASA (Å^2^) Subunit A**	**Dimer ASA (Å^2^) Subunit B**	**Monomer ASA (Å^2^) Subunit A**
C13 (cTPI)	2.6	2.8	129.3
C67 (cTPI)	0.1	0.0	0.1
C127 (cTPI)	0.0	0.1	0.0
C218 (cTPI)	6.7	2.6	6.7
C15 (pdTPI)	2.8	3.3	127.2
C89 (pdTPI)	8.2	8.5	8.2
C127 (pdTPI)	0.0	0.0	0.0
C144 (pdTPI)	0.8	0.1	0.8

**Table 3:** **Accessible thiols present in wild-type and mutants AtTPIs determined by DTNB**.

**cTPI enzyme version**	**Accessible thiols (-SH)/monomer**	**pdTPI enzyme version**	**Accessible thiols (-SH)/monomer**
WT	2.0 ± 0.12	WT	1.6±0.13
C13S	1.1 ± 0.07	C15S	0.1±0.02
C218S	1.0 ± 0.08	C89S	0.9±0.1
C13S/C218S	0.9 ± 0.06	C15S/C89S	2.0±0.21

### Redox modulation of AtTPIs

**Figure 4:**
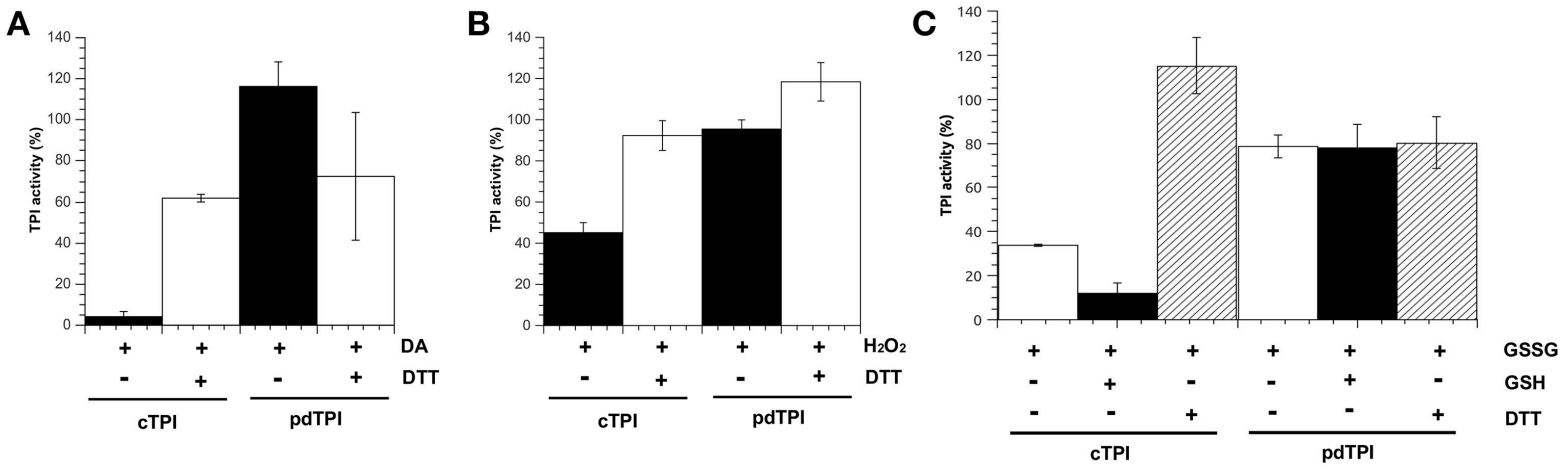
**Modulation of AtTPI enzymatic activity by redox agents**. AtTPIs enzymatic activities after treatment for 2 h at 37°C with diverse redox agents in comparison to the enzymatic activity of untreated proteins. **(A)** Incubation of cTPI and pdTPI in the presence of 1 mM diamide (DA) (black bars). The reversibility of the reaction was tested by incubating with 20 mM DTT (white bars). **(B)** Incubation of cTPI and pdTPI in the presence of 0.2 mM H_2_O_2_ (black bars). The reversibility of the reaction was tested by incubating with 20 mM DTT (white bars). **(C)** Incubation of cTPI and pdTPI in the presence of 2.5 mM GSSG (black bars). The reversibility of the reaction was tested by incubating with 2.5 mM GSH(white bars) or 20 mM DTT (white bars with black lines).

The reduction-oxidation (redox) state affects the activity of numerous enzymes in *A. thaliana* and other plant systems (Ito et al., 2003; Zaffagnini et al., 2007, 2013; Sun et al., 2012; Michelet et al., 2013). For example, the activity of glyceraldehyde 3-phosphate dehydrogenase (GAPDH) is inhibited by GSSG, H_2_O_2_ and diamide (Holtgrefe et al., 2008; Zaffagnini et al., 2013) and α-amylase and starch synthase, are regulated by disulfide-bond formation (Seung et al., 2013; Skryhan et al., 2015). Due to the central role of AtTPIs in carbon metabolism, we tested whether redox agents may have an effect on cTPI and pdTPI catalytic activities. Diamide (DA) is a thiol-oxidizing agent able to modify exposed cysteines (Kosower and Kosower, 1995). We found that the addition of 1 mM of DA inhibited cTPI activity to less than 5% of the untreated level, a response also observed in yeast TPI (Ralser et al., 2006). Inhibition could be reversed by the addition of 20 mM DTT. On the other hand, pdTPI was not inhibited by addition of DA (Figure 4A) but addition of DTT lead to a decrease in the activity of this enzyme (Figure 4A). The addition of 200 μM H_2_O_2_ decreased the activity of cTPI to approximately 40% of its untreated activity, and this result could be reversed by the addition of 20 mM DTT whereas pdTPI activity was 90% in the presence of 200 μM H_2_O_2_ with respect to the untreated activity (Figure 4B). The addition of 2.5 mM GSSG drastically decreased the activity of cTPI to ~30% of its untreated activity, but only produced a slight reduction in pdTPI activity (Figure 4C). The inhibitory effect for cTPI was not reversed by the addition of 2.5 mM GSH, however the addition of 20 mM DTT reversed the inhibitory effect of GSSG on cTPI activity to untreated activity levels (Figure 4C). The responses to oxidative agents observed in pdTPI are similar to those observed in CrTPI, where the addition of H_2_O_2_ did not produce a significant effect on enzymatic activity and GSSG addition only induced a slight decrease in activity (Zaffagnini et al., 2014), in contrast cTPI is highly susceptible to thiol modifications induced by DA and GSSG.

### Accessibility of cysteine thiols (-SH)

Our structural analysis indicates that residues C13 and C218 of cTPI and residues C15 and C89 of pdTPI are solvent-exposed. In order to test whether those residues are targets for redox modification, we produced two single mutants of cTPI: cTPI-C13S and cTPI-C218S, and a double mutant where both cysteines were replaced by serine residues. We also generated two single mutants for pdTPI (pdTPI-C15S and pdTPI-C89S) and a double mutant including both substitutions. In order to determine whether mutation of the exposed cysteines influenced AtTPI oligomerization, we measured their oligomeric state using analytical gel filtration. cTPI-C13S, cTPI-C13S/C218S, pdTPI-C15S, and pdTPI-C89S mutants, present a molecular mass that corresponds to dimeric molecules, whereas the double mutant pdTPI-C15S/C89S showed a mixture of dimers and monomers, suggesting that the combined effect of the double mutant destabilizes the pdTPI dimer (Figure S5). Thiol accessibility for mutant AtTPIs was determined using DTNB. Point mutants cTPI-C13S and cTPI-C218S decrease the number of accessible -SHs to one indicating that residues cTPI-C13 and cTPI-C218 are accessible to DTNB (Table 3). A total loss of the accessible cysteines is expected for the double mutant cTPI-C13S/C218S, however this double mutant also presents one accessible cysteine per monomer, suggesting that the double mutant induces partial unfolding and partially exposes buried cTPI cysteines. Point mutant pdTPI-C15S did not present DTNB reactivity, indicating that this residue accounts for the exposed -SH groups. Point mutant C89S decreases the number of accessible thiols from 1.6 to 0.9, this reduction indicates a partial contribution of this residue in the accessible -SH groups present in pdTPI. Surprisingly the double mutant pdTPI-C15S/C89S presents two exposed cysteines per monomer. This phenomenon can be explained by complete pdTPI unfolding and the exposure of the -SH of the buried residues pdTPI-C127 and pdTPI-C144. Our results suggest that residues C13 and C218 of cTPI could be targets for redox modulation in cTPI, while C15 appears as the only possible target in pdTPI.

### Effect of mutation of cysteine residues on TPI activity

Contrasting results have been obtained for the role of cysteines in the enzymatic activity of TPIs. For example, substitution of C13 to aspartate causes a 7-fold reduction in the enzymatic activity of *P. falciparum* (Maithal et al., 2002), while in *T. brucei* a mutation of C14 to serine had no effect (Perez-Montfort et al., 1999). With these contrasting examples, it is difficult to predict the response of AtTPIs to cysteine substitutions. Thus, enzymatic assays directed at testing the effect of mutations on AtTPI activity were performed using 1 mM G3P as a substrate. All data were calculated taking the activity of wild-type AtTPIs as a reference value of 100%. This assay revealed a substantial decrease in enzymatic activity for cTPI mutants where the remaining TPI activities were below 3.5% for single and double cTPI mutants (Table 4). In the case of pdTPIs, the catalytic activities of C15S and C15S/C89S mutants were substantially inhibited, with remaining activities below 0.25%. In contrast, pdTPI-C89S displayed 66.6% of the wild-type activity. These data suggest that residues cTPI-C13, cTPI-C218, and pdTPI-C15 are necessary for AtTPI enzymatic activity, whereas substitution of residue pdTPI-C89 by serine is tolerable (Table 4).

**Table 4:** **Relative activity of AtTPI mutants vs. their wild-type counterparts**.

**Enzyme**	**Activity vs. WT (%)**	**Enzyme**	**Activity vs. WT (%)**
cTPI WT	100 ± 2.01	pdTPI WT	100±3.26
cTPI C13S	1.52 ± 0.05	pdTPI C15S	0.25±0.01
cTPI C218S	0.70 ± 0.03	pdTPI C89S	66.66±0.50
cTPI C13S/C218S	2.04 ± 0.01	pdTPI C15S/C89S	0.04±0.00

### Effect of the sulfhydryl reagent MMTS on AtTPI activity

**Figure 5:**
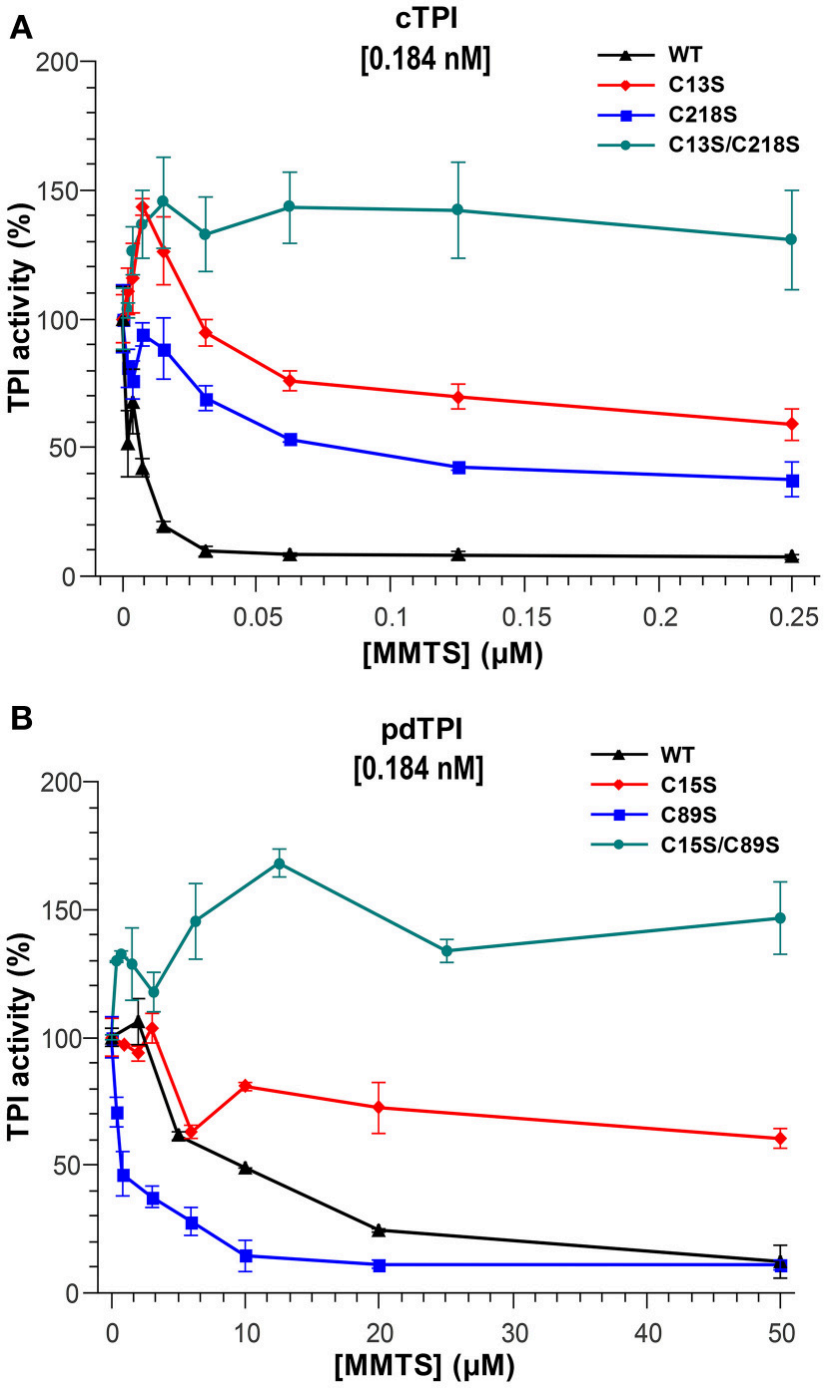
**Effect of the sulfhydryl reagent MMTS on AtTPI catalytic activity**. **(A,B)** Effect of the addition of increasing concentrations of MMTS on the activity of wild-type and mutant AtTPIs. **(A)** Illustrates the effect on cTPI activity, and **(B)** the effect on pdTPI activity. The concentration of MMTS in cTPI varies from 0 to 0.25 μM, whereas the MMTS concentration used for pdTPI is from 0 to 50 μM. The results are expressed as a percentage of the activity with respect to the untreated protein sample. Protein concentrations are indicated.

Sulfhydryl reagents such as methyl methane thiosulfonate (MMTS) decrease the catalytic activity of TPIs by conjugating to thiol accessible cysteines (Perez-Montfort et al., 1999; Enriquez-Flores et al., 2008). We hypothesized that single cysteine AtTPI point mutants should increase the amount of sulfhydryl conjugating agents necessary to achieve TPI inhibition and that a mutant without accessible cysteines should be resistant to MMTS inhibition. The inhibitory effect of MMTS on AtTPI activity exhibits clear differences in relation to the different isoforms of the enzymes. The activity of cTPI, present at a concentration of 0.184 nM (10 ng/ml), decreases by 90% after treatment with 31.25 nM of MMTS. pdTPI is also inhibited to 90% of its untreated values, but in the presence of 50,000 nM MMTS. Thus, the molar ratio to inhibit by 90% the activities of cTPI and pdTPI are approximately 142 and 271,739- times respectively. Inhibition profiles also showed that MMTS at a concentration of 2.5 μM does not inhibit pdTPI activity, whereas 0.025 μM MMTS, corresponding to a 135-fold molar excess of inhibitor, is sufficient to completely inhibit cTPI. Thus, pdTPI is at least 100-fold more resistant than cTPI to MMTS inhibition. cTPI single point mutants were partially inhibited by MMTS, displaying activities of 48% for cTPI-C13S and 31% for cTPI-C218S with respect to the activity of the untreated mutants. The MMTS concentration necessary to inhibit 50% of cTPI-218S was 0.125 μM and at this concentration the cTPI-C13S mutant presents 70% of its activity with respect to the untreated protein. As expected the enzymatic activity of the double mutant cTPI-C13S/C218S was not affected by the addition of MMTS. In contrast to the nanomolar MMTS concentrations needed to inhibit cTPI, pdTPI presented 50% of its enzymatic activity at concentrations greater than 10 μM (Figure 5). Mutation pdTPI-C89S is more susceptible to MMTS inhibition, possibly due to a decrease in protein stability and an increase in the accessibility of pdTPI-C15 (Figure 5). As expected, the double mutant C15S/C89S was insensitive to the addition of the inhibitor (Figure 5).

### Effects of GSSG on cTPI and pdTPI activity and dimer dissociation

**Figure 6:**
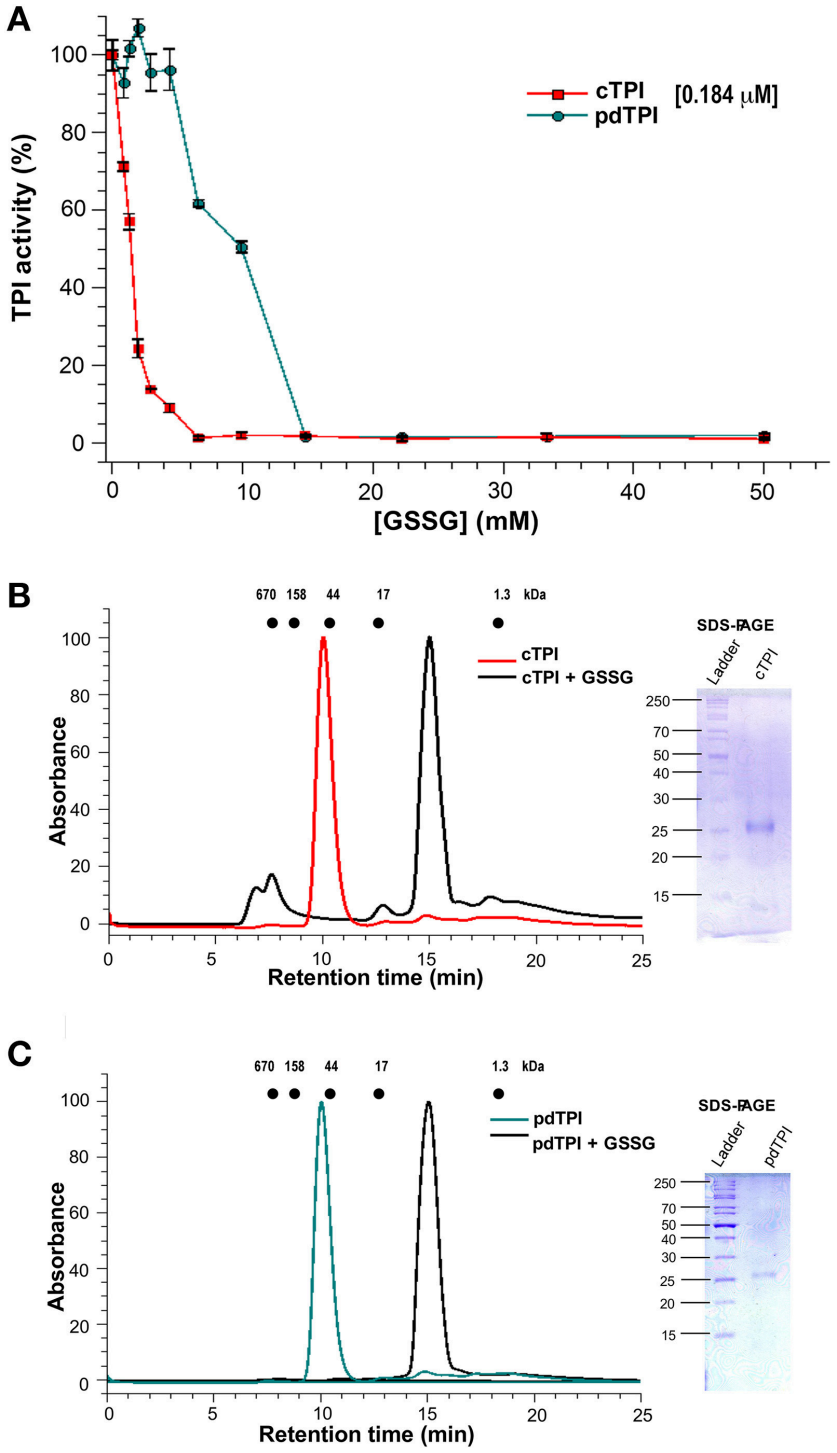
**Differential effect of GSSG on the enzymatic activity of AtTPIs and dimer stability. (A)** Effect of GSSG on the enzymatic activity of cTPI (red) and pdTPI (green) after 3 h of incubation at 25°C with increasing concentrations of oxidized glutathione (0–25 mM). The results are expressed as a percentage of the activity with respect to the untreated protein sample. **(B,C)** Effect of addition of GSSG on the oligomerization of AtTPIs. **(B)** Size-exclusion profile showing the effect of the addition of GSSG on cTPI dimer stability. The non-derivatized enzyme is shown in red, whereas the GSSG incubated enzyme is shown in black. The SDS-PAGE shows the molecular weight of collected fractions of the cTPI-GSSG peak. **(C)** Size-exclusion profile showing the effect of the addition of GSSG on the pdTPI oligomeric profile. The non-derivatized enzyme is shown in green and the GSSG treated enzyme is shown in black. The SDS-PAGE shows the molecular weight of collected fractions of the pdTPI-GSSG peak. The elution profiles for MW standards; thyroglobulin (670 kDa), γ-globulin (158 kDa), ovalbumin (44 kDa), myoglobin (17 kDa), and vitamin B12 (1.35 kDa) are represented as dots.

Glutathionylation regulates the activity of several plant proteins such as glyceraldehyde-3-phosphate dehydrogenase, glucose-6-phosphate dehydrogenase, and thioredoxin (Wenderoth et al., 1997; Gelhaye et al., 2004; Bedhomme et al., 2012). TPI s also present a range of responses to glutathionylation, for example, in chicken and *C. reinhardtii* the addition of GSSG induces only slight changes in TPI activity (Tang et al., 1990; Zaffagnini et al., 2014), whereas the addition of GSSG inhibits the enzymatic activity of the cTPI from *A. thaliana* (Ito et al., 2003). To understand the role of glutathionylation in AtTPI activity, we performed a kinetic assay using various concentrations of GSSG. cTPI displayed higher sensitivity to GSSG than its plastid counterpart (Figure 6A) since cTPI activity decreased with all GSSG concentrations tested. At a GSSG concentration of 0.87 mM, using cTPI at 0.184 μM (10 μg/ml), enzymatic activity decreased to ~70% with respect to the activity of the untreated cTPI and was completely inhibited at concentrations above 6.5 mM. In contrast the addition of GSSG did not induce a significant effect on pdTPI activity in a range of concentrations from 0.87 to 4.3 mM. However, pdTPI enzymatic activity decreased to 62 and 50% of the untreated activities in the presence of 6.5 and 9.9 mM of GSSG respectively. Total inhibition of pdTPI was achieved at 14.9 mM.

In order to understand the mechanism of AtTPI regulation by glutathione, we determined the molecular weight of derivatized AtTPIs with glutathione by analytical size-exclusion chromatography. For both AtTPIs, the addition of 5 mM GSSG in combination with overnight incubation at room temperature induced a displacement of the protein peak to fractions with lower molecular weight. The protein peaks from the AtTPIs subject to GSSG treatment displayed an apparent MW of approximately 10 KDa that is lower than the expected MW of a TPI monomer (27 kDa). However, a SDS-PAGE of the recovery fractions indicated the presence of protein bands with molecular weights of 27 kDa (Figures 6B,C). These fractions did not present catalytic activity.

### Identification of glutathionylation sites

**Figure 7:**
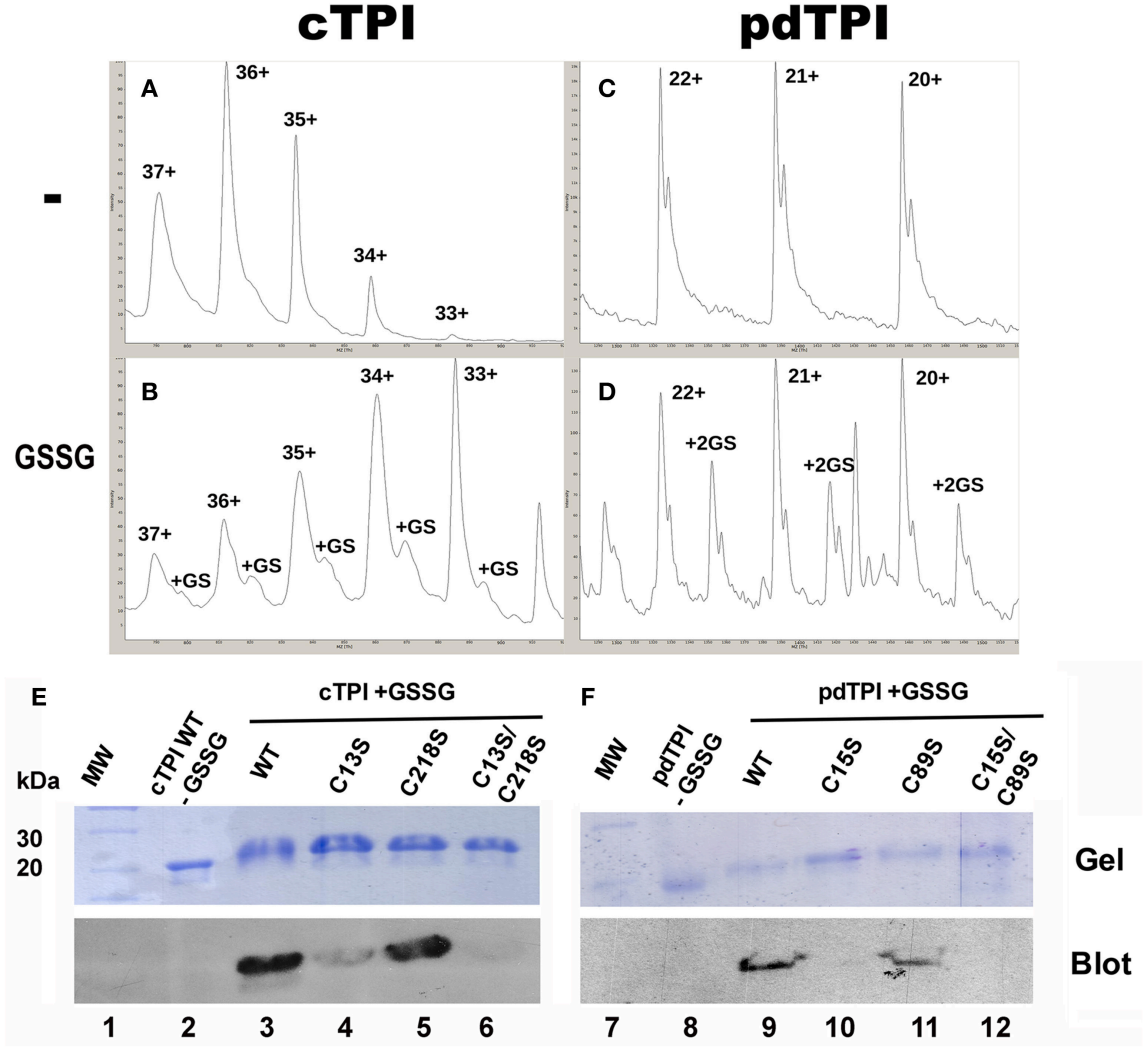
**Glutathionylation of AtTPIs**. **(A)** Mass spectrum for untreated cTPI. **(A)** and pdTPI **(C)**, Mass spectrum of cTPI treated with 0.25 mM GSSG, demonstrating a mass shift corresponding to one glutathione **(B)**. Mass spectrum of pdTPI treated with 25 mM GSSG, congruent with two covalently bound glutathiones **(D)**. **(E,F)** Identification of *Arabidopsis* cTPI and pdTPI glutathionylation by Western blot. Purified AtTPI proteins were incubated in the presence of GSSG and transferred onto a nitrocellulose membrane for Western blot analysis. In the case of cTPI, glutathionylation was detected for the wild-type enzyme and the single C15 and C218 mutants, whereas no signal was detected for the double C15-C218 mutant. For pdTPI, glutathionylation was detected for the wild-type enzyme and the C89S mutant, but not for the single C13S and the double C89S-C13S mutant.

We evaluated the formation of glutathionylated AtTPIs *in vitro* by incubating cTPI and pdTPI with GSSG concentrations ranking from 0.25 to 25 mM and analyzing the reaction products by mass spectrometry. In a top-down experiment we aimed to observe mass shifts in AtTPIs that would correspond to the number of glutathionylation sites. The average theoretical molecular mass of recombinantly expressed cTPI is 29,117.25 Da, whereas the average molecular mass for pdTPI is 29,098.98 Da. The addition of one molecule of GSH would add a mass of 305.07 Da and the addition of two GSH molecules would add a mass of 612.61 Da to each recombinant protein. We subjected the untreated protein samples to electrospray Ionization mass spectrometry (ESI-MS), and the average calculated molecular masses for cTPI and pdTPI were 29,205.15 and 29,096.83 Da respectively (Figures 7A,C). Upon GSSG treatment the molecular mass of cTPI increased 295 Da, indicating that one GSH molecule binds to cTPI (Figure 7C). This increase in molecular mass was only observed at a GSSG concentration of 0.25 mM. At higher GSSG concentrations, cTPI precipitates on dialysis with water. On the other hand, pdTPI presented no variation in mass until the protein was subjected to incubation with 25 mM of GSSG (Figure 7D). With this treatment we observed an increase in mass of 612.61 Da indicating that pdTPI conjugates with two molecules of GSH.

To further investigate the identity of AtTPI residues that could be glutathionylated, wild-type and mutant versions of cTPI and pdTPI were subject to S-glutathionylation *in vitro* with the goal being to detect this modification by Western blot using a biotinylated glutathione-specific antibody (BioGSSG). This antibody was able to detect the presence of glutathionylated residues in cTPI and pdTPI treated with GSSG (Figures 7E,F). A Western blot signal was also detected in cTPI-C13S and cTPI-C218S individual mutants. However, the intensity of the signal revealed that residue cTPI-C13 is more prone to be glutathionylated than cTPI-C218. As expected, the glutathionylation signal was absent on the cTPI-C13S/C218S double mutant, indicating that in cTPI only residues C13 and C218 are glutathionylated and are potential targets of glutathionylation *in vivo* (Figures 7E,F). In the case of pdTPI mutants, a Western blot signal was not detected for the pdTPI-C15S mutant, whereas the intensity of the signal for pdTPI-C89S was similar to that of the wild-type pdTPI (Figures 7E,F). In this case, our data suggest that C15 is the main target of glutathionylation in pdTPI.

## Discussion

Crystal structures of AtTPIs allowed us to establish a direct comparison between cTPI and pdTPI isoforms. Both AtTPIs fold into a (β-α)_8_ barrel in which loop 1, loop 2, loop 4, loop 8 and distinctively loop 3 assemble the dimer interface and loop 6 is present in an open conformation. In order to understand whether AtTPIs can be subject to redox-modifications we analyzed the structural context of their cysteines. Both cTPI and pdTPI contain 4 cysteines in their primary structure: cTPI-C13, cTPI-C67, cTPI-C127, cTPI-C218 and pdTPI-C15, pdTPI-C89, pdTPI-C127, pdTPI-C144. Residues pdTPI-C127 and cTPI-C127, cTPI-C67, and pdTPI-C144 are buried and point toward the hydrophobic core of the protein and were not studied in this work. The role of cysteines located in structural positions equivalent to cTPI-C13 (or pdTPI-C15) and C127 has been widely studied in various organisms, mainly in relationship to enzymatic activity and protein stability (Reyes-Vivas et al., 2001; Maithal et al., 2002; Gonzalez-Mondragon et al., 2004; Samanta et al., 2011). A residue analogous to cTPI-C218 decreases enzymatic activity upon derivation in *G. lamblia* (Enríquez-Flores et al., 2011). AtTPI crystal structures and numerous other evidence indicate that residues cTPI-C13, cTPI-C218, pdTPI-C15, and pdTPI-C89 are solvent exposed, however the thiol group of pdTPI-C89 points toward the hydrophobic core. DTNB conjugation indicates that cTPI presents 2 accessible cysteines suggesting that both cTPI-C218 and cTPI-C13 are possible targets of redox agents. In the case of pdTPI, DTNB conjugation indicates that this protein contains 1.6 cysteines per monomer, this number can be associated with local structural fluctuations or breathing motions near buried thiol groups. Previously it has been shown that residue K84 in yeast TPI exhibits breathing motions (Massi et al., 2006), since this residue is located at an analogous structural position near pdTPI-C89 it is possible that structural transitions may transiently expose the thiol group of C89 to the solvent.

**Figure 8:**
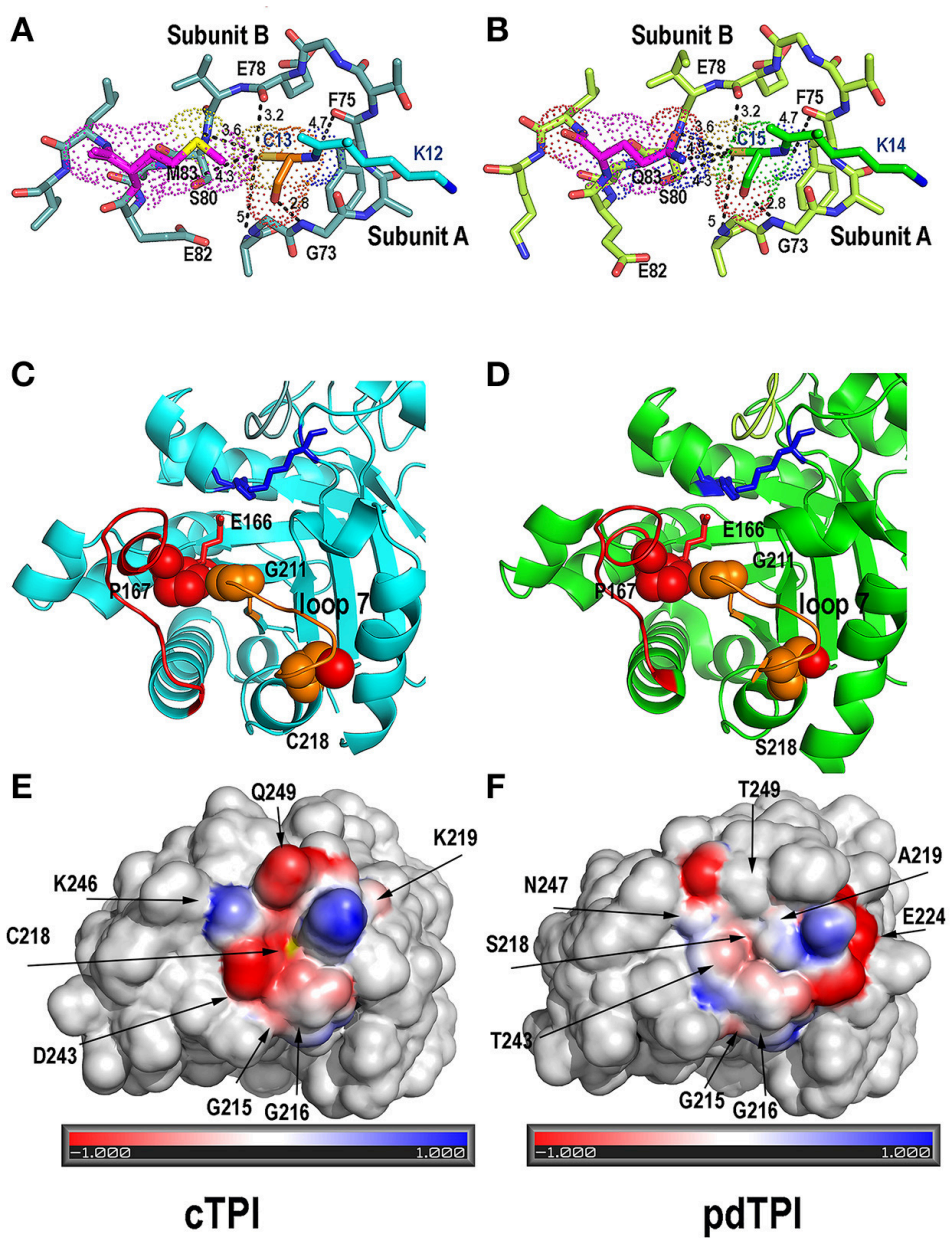
**Structural rationale for cTPI susceptibility and pdTPI resistance to redox agents. (A,B)** Close-up of the interface between loop 1 cysteine (cTPI-C13 or pdTPI-C15) and loop 3 from the neighboring subunit. Loop 1 cysteine is displayed in a ball-stick representation and colored in orange (cTPI-C13) or green (pdTPI-C15). A conserved set of interactions between residues G73, S89, E78, and F75 of the neighboring AtTPI subunit is displayed. cTPI-C-13 forms a hydrophobic interaction with cTPI-M13, whereas pdTPI-C15 has the potential to form a H-bond with pdTPI-Q83. **(C,D)** Close Up of the α-helix 7 AtTPIs showing residues cTPI-C218 **(C)** and pdTPI-S218 **(D)** in a surface representation. The thiol and hydroxyl groups of cTPI-C218 and pdTPI-S218 are colored in red. Both residues are located near residue G211 of the YGGS motif that makes contact with residue P167 located immediately before the catalytic E166. The structural localization of cTPI-C218 predicts that alterations in its integrity maybe transduced to the catalytic E166. **(E,F)** Electrostatic potentials near residues cTPI-C218 and pdTPI-C218. Positively charged amino acids are colored in blue, negatively charged amino acids in red. The thiol group of cTPI-C218 is colored in yellow.

cTPI is inhibited by diamide, H_2_O_2_, and GSSG at a concentration of 2.5 mM, in contrast pdTPI is resistant to inhibition by these redox agents (Figure 4). The responses of pdTPI to oxidative agents are similar to those observed in CrTPI, where the addition of H_2_O_2_ did not produce a significant effect on enzymatic activity and addition of GSSG only induced a slight decrease in activity (Zaffagnini et al., 2014). In order to investigate a role for the putative redox response of AtTPI cysteines in regulating enzymatic activity, we carried out kinetic studies in which solvent accessible cysteines were mutated to serine. We reasoned that serine mutants would be the less perturbing as the only difference between these amino acids is that the thiol group is replaced by a hydroxyl group. Surprisingly substitutions of cTPI-C13, cTPI-C218, and pdTPI-C15 to serine decrease the enzymatic activity by nearly 100-fold of their corresponding wild-type AtTPI activity. Crystal structures show that pdTPI-C15 is contacted by hydrogen bonds with side chains of residues E78, S80, and Q83, and the main chains of F75 and G73 of the neighboring monomer, whereas cTPI-C13 makes the same interactions as pdTPI-C15 but residue Q83 is replaced by M83 (Figures 8A,B). Serine substitutions of cysteines located in a structurally equivalent position of cTPI-C13 and pdTPI-C15 in *T. brucei* and *T. cruzi* (*T. brucei*-C14S, *T. cruzi*-C15S) have no significant effect on catalytic activity (Hernández-Alcántara et al., 2002; Cabrera et al., 2008). However, substitution of those residues to hydrophobic and charged amino acids decreased enzymatic activity by more than 1000-fold (Hernández-Alcántara et al., 2002; Banerjee et al., 2011). In TPIs the epsilon amino group of the catalytic lysine guides the transfer of the hydroxyl proton during G3P and DHAP interconversion and this amino acid has to be properly oriented for catalysis (Jogl et al., 2003). The substitution of Cys to Ser decreases the ASA by 24 A^2^, suggesting that the tight packing of cTPI-C13 and pdTPI-C15 and the substitution of a hydrophobic cysteine for a polar serine decreases catalytic activity by altering the geometry of the catalytic lysine (Nagano et al., 1999). Residue cTPI-C218 is located on α-helix 7, distant from the active site and the dimer interface (Figure 8). cTPI-C218S mutant exhibits a decrease in activity by more than 140-fold in comparison to wild-type cTPI activity. The localization of residue cTPI-C218 resembles the localization of residue C222 in the *G. lamblia* TPI. Point mutants of this structurally analogous cysteine to alanine and lysine decrease catalysis by 2- and 159-fold respectively, whereas derivatization induces complete TPI inactivation (Enríquez-Flores et al., 2011; Hernández-Alcántara et al., 2013). cTPI-C218 interacts with residue G211 that contacts residues located before the catalytically essential E166. Residue G211 is part of the conserved YGGS motif and mutations of these amino acids dissociate the closed TPI conformation and decrease catalysis by more than 2000-fold (Sampson and Knowles, 1992).

TPIs present a wide range of responses to thiol-specific conjugation reagents (Perez-Montfort et al., 1999; Téllez-Valencia et al., 2002). cTPI is at least 100 times more susceptible to MMTS conjugation than pdTPI. This phenomenon resembles the difference in inhibition observed between *T. cruzi* and *T. brucei* TPIs. Specifically, complete *T. cruzi* TPI inhibition (protein treated at a concentration of 18 nM) is achieved at a concentration of 10 μM MMTS, whereas complete *T. brucei* TPI inhibition is achieved at 0.4 mM of MMTS. This data indicates that approximately a 555 and 22,222 molar excess of MMTS is necessary to inhibit TPIs from *T. cruzi* and *T. brucei* respectively and that *T. cruzi* TPI is approximately 40 times more susceptible to MMTS than *T. brucei*. Our results indicate that the molar ratio of MMTS/enzyme necessary to inhibit cTPI is of 142-fold molar excess of MMTS and the molar ratio to inhibit pdTPI is 271,739-fold of MMTS. The latter indicates a differential of approximately 1910 times in the susceptibility of cTPI with respect to pdTPI to MMTS inhibition. Although the need for a 271,739-fold molar excess of MMTS to achieve inhibition may appear excessive, TPIs from yeast and rabbit present no inactivation responses to concentrations up to 2000-fold molar excess of MMTS (Gómez-Puyou et al., 1995).

Glutathionylation is a protective mechanism that prevents the irreversible oxidation of exposed specific cysteines to cysteine sulfinic or sulfonic acid (Zaffagnini et al., 2012a,b). Nevertheless, if a cysteine is involved in catalysis, glutathionylation would inhibit enzymatic activity. Seminal work by Ogawa's laboratory indicated that cTPI at a concentration of 18.52 nM (5 ng/μl) is inhibited by approximately 70% of its untreated activity by 2.5 mM of GSSG after 3 h of incubation (Ito et al., 2003). This data indicates that GSSG must be present at a molar excess of 135,000 times to achieve efficient cTPI inhibition. In our hands, cTPI incubated at a concentration of 184 nM is completely inhibited by 6.5 mM of GSSG, indicating that a molar excess of 35,326 times of GSSG is sufficient to inactivate cTPI. pdTPI presents complete inhibition at a concentration of 14.9 mM GSSG. Because the derivatization of cysteines structurally analogous to cTPI-C13 and pdTPI-C15 induces monomerization in *P. falciparum* and *E. histolytica* TPIs, we were curious to investigate the oligomeric state of AtTPIs upon GSSG derivatization (Maithal et al., 2002; Rodríguez-Romero et al., 2002). We found that at a concentration of 5 mM of GSSG and overnight incubation, GSSG induces dimer dissociation of AtTPIs (Figures 6B,C). This observation indicates that glutathionylation decreases catalysis by altering the orientation of catalytic amino acids and by promoting the emergence of non-catalytic monomeric AtTPIs.

ESI-MS data indicate that cTPI and pdTPI are conjugated with one and two molecules of GSH respectively, in contrast Western blot analysis indicate that cTPI binds to one GSH molecule and pdTPI to two molecules (Figure 7). In the ESI-MS analysis, cTPI conjugation is achieved at 0.25 mM of GSSG and at higher concentrations the protein precipitates upon its transfer to water. In contrast, pdTPI conjugation is only achieved at 25 mM GSSG and at lower concentrations no derivatization is observed. Upon GSSG derivatization and Western blot analysis of wild-type cTPI and mutants, cTPI-C218S presents a band that exhibits the same intensity as wild-type cTPI, whereas cTPI-C13S exhibits a faint band. The fact that cTPI-C13 is the main target of glutathionylation indicates that at lower GSSG concentrations this residue should be conjugated. Higher concentrations of GSSG induce protein aggregation making it impossible to analyze cTPI glutathionalytion by ESI-MS. This is congruent with the notion that in most TPIs characterized to date dimer dissociation induces monomer unfolding (Mainfroid et al., 1996; Rietveld and Ferreira, 1998; Chánez-Cárdenas et al., 2002; Pan et al., 2004). Western blot analysis of wild-type pdTPI and its related mutants indicate that pdTPI-C15S is unable to react with glutathione, whereas pdTPI-C89S presents similar reactivity as wild-type pdTPI. In this case, the data suggest that residue pdTPI-C15 is the main target of glutathionylation. Since pdTPI reacts only at high concentrations of GSSG it is possible that conformational transitions of residue C89 induced glutathione derivatization.

We have shown that residues cTPI-C13, cTPI-c218, and pdTPI-C15 are essential for catalysis as these residues react with glutathione and their mutations substantially decrease enzymatic activity. The remaining question is how pdTPI achieves resistance whereas cTPI presents susceptibility to oxidative agents? Crystallographic data shows that an AtTPI cysteine located in loop 1 (pdTPI-C15 or cTPI-C13) interacts with a conserved set of residues from loop 3 of the neighboring subunit (Figures 8A,B). From these loop 3 residues, the only interaction that is different between AtTPIs, is that in cTPI residue M83 makes a hydrophobic interaction with cTPI-C13, whereas in pdTPI residue Q83 makes a H-bond with pdTPI-C15. TPIs from T. *cruzi* and *T. brucei* share 73.6% of amino acid sequence identity, however loop 1 cysteine of *T. cruzi* is more susceptible to conjugation (Reyes-Vivas et al., 2001). This difference in reactivity is explained by the character of neighboring amino acids which modulate the pKa of loop 1 cysteines (García-Torres et al., 2011). Our structural data suggest that the H-bond interaction of pdTPI-C15 to pdTPI-Q8 is responsible for decreased reactivity in pdTPI-C15 (Figures 8A,B). This hypothesis is congruent with the observation that a conserved set of H-bonds participate in thiolate formation in fission yeast DJ-1 (Madzelan et al., 2012). The other cysteine residue that may account for redox modifications is cTPI-c218. Proteomic studies from mice and *Homo sapiens* indicate that TPIs from those organisms are nitrosylated at a residue structurally analogous to cTPI-218 (Doulias et al., 2010; Fares et al., 2011; Murray et al., 2012). Residue cTPI-C218 is the most exposed cysteine in cTPI, in contrast pdTPI presents a serine at this position (pdTPI-S218). cTPI-C218 and pdTPI-S218 are surrounded by charged amino acids. However, cTPI-C218 is located before a lysine, whereas pdTPI-S218 is positioned before an alanine indicating that the epsilon amino group of cTPI-K219 may contribute to the reactivity of cTPI-C218 (Figures 8C–F).

In conclusion, our data indicate that the duplicated TPI gene from *A. thaliana* has suffered two evolutionary fates: the cTPI isoform is susceptible to redox agents, whereas the pdTPI isoform is resistant. Structural studies lead us to postulate that subtle changes in amino acid sequence such as the substitution of cTPI-M83 for pdTPI-Q83 and cTPI-C218-K219 for pdTPI S218-A219 are responsible for the differential responses of the AtTPI isoforms to oxidative agents. Our studies indicate that AtTPI glutathionylation has two consequences: firstly, it is a process that protects AtTPIs from irreversible oxidation and secondly it decreases catalysis by perturbing the active site or altering the dimer-monomer equilibrium. The physiological role of a decrease in TPI activity may be related to a re-routing of the carbon flux during oxidative stress. This re-routing of the metabolic flux is a conserved feature in other eukaryotic organisms (Ralser et al., 2006, 2007).

## Accession number

The atomic coordinates and structural factors of AtTPIs are publically available at the Protein Data Bank with accession codes 4OBT and 4OHQ.

## Author contributions

LB: conceived the idea and data analysis; LL-C: conceived the idea, performed experiments, data analysis, and wrote manuscript; PJ-S: perform experiments and data analysis; NB-T: perform experiments and data analysis; CT-A: perform experiments and data analysis; CD-Q: perform experiments and data analysis; SL-G: data analysis; RW: conceive and perform experiments and data analysis.

### Conflict of interest statement

The authors declare that the research was conducted in the absence of any commercial or financial relationships that could be construed as a potential conflict of interest.

## Supplementary material

Figure S1 **Amino acid sequence alignment of ***A. thaliana*** TPIs. (A)** Sequence alignment between cTPI and the mature sequence of pdTPI illustrates that both enzymes share more than 60% amino acid identity. **(B)** Structural representation of PdTPI showing one monomer in carton representation and one monomer in ribbon representation. Secondary structure elements determined for their function (loop 3, YGGF motif, phosphate binding motif, loop 6). Catalytic amino acids are indicated in the primary and tertiary structure.

Figure S2 **Purification, oligomeric state, and crystals from AtTPIs. (A)** Eluate of cTPI (lane 2), pdTPI (lane 3), and sp-pdTPI (lane 4) after IMAC purification. Lane 1 indicates the MW ladder. **(B)** Chromatogram showing the elution profile of both AtTPIs. **(C)** Crystals obtained from cTPI and pdTPI used for X-ray diffraction and further resolution of both structures.

Figure S3 **Structural comparison between AtTPIs and CrTP1**. Superposition of the three crystal structures from photosynthetic organisms showing their active site and dimer interface. The catalytic residues Lys, His, and Glu are in a stick representation. Molecule B is presented to highlight the formation of the dimeric species and the interface.

Figure S4 **Crystal structures of AtTPIs. (A,B)** Surface representation of the crystal structures of cTPI and pdTPI (green and cyan, respectively) showing the localization of their solvent exposed cysteines. **(C)** Structural localization of residue C13 on the cTPI crystal structure at the dimer interface. **(D)** Structural localization of residue C218 on the cTPI crystal structure. This residue is located at a distant position from the active site or the dimer interface. **(E)** Structural localization of C15 on the pdTPI crystal structure. As in cTPI-C13, this residue is part of the dimer interface. **(F)** Structural localization of C89 residue on the pdTPI crystal structure. The sulfhydryl group of pdTPI-c89 points toward the hydrophobic core.

Figure S5 **Single and double mutations alter the oligomeric state of AtTPIs. (A,B)** Size-exclusion chromatography profiles showing the oligomeric state of single and double mutants of cTPI and pdTPI. The oligomeric state of cTPI is resistant to the effect of single and double mutants **(A)**, whereas a double mutant of pdTPIC15S-C89S reduces the fraction of dimeric protein **(B)**.

Table S1 **Crystallographic data collection parameters and statistics**.
