# Supplementary figures and images for: Structural Basis for Redox Regulation of Cytoplasmic and Chloroplastic Triosephosphate Isomerases from Arabidopsis thaliana
Source: Front Plant Sci. 2016 Dec 6;7:1817. doi: 10.3389/fpls.2016.01817 (PMC5138414; doi:10.3389/fpls.2016.01817)

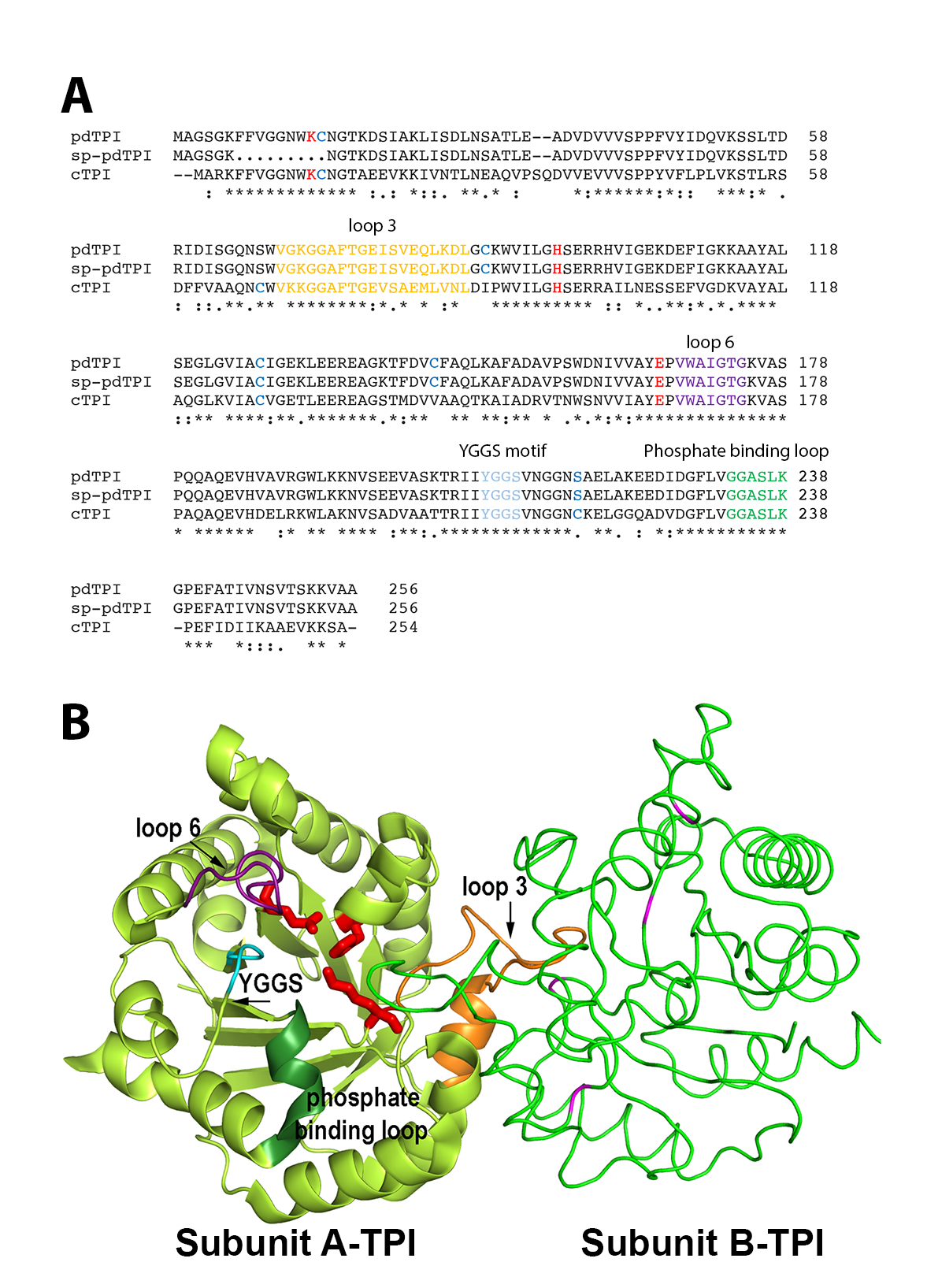

Supplement: Figure S1 — Amino acid sequence alignment of A. thaliana TPIs. (A) Sequence alignment between cTPI and the mature sequence of pdTPI illustrates that both enzymes share more than 60% amino acid identity. (B) Structural representation of PdTPI showing one monomer in carton representation and one monomer in ribbon representation. Secondary structure elements determined for their function (loop 3, YGGF motif, phosphate binding motif, loop 6). Catalytic amino acids are indicated in the primary and tertiary structure. [file Image1.TIF]

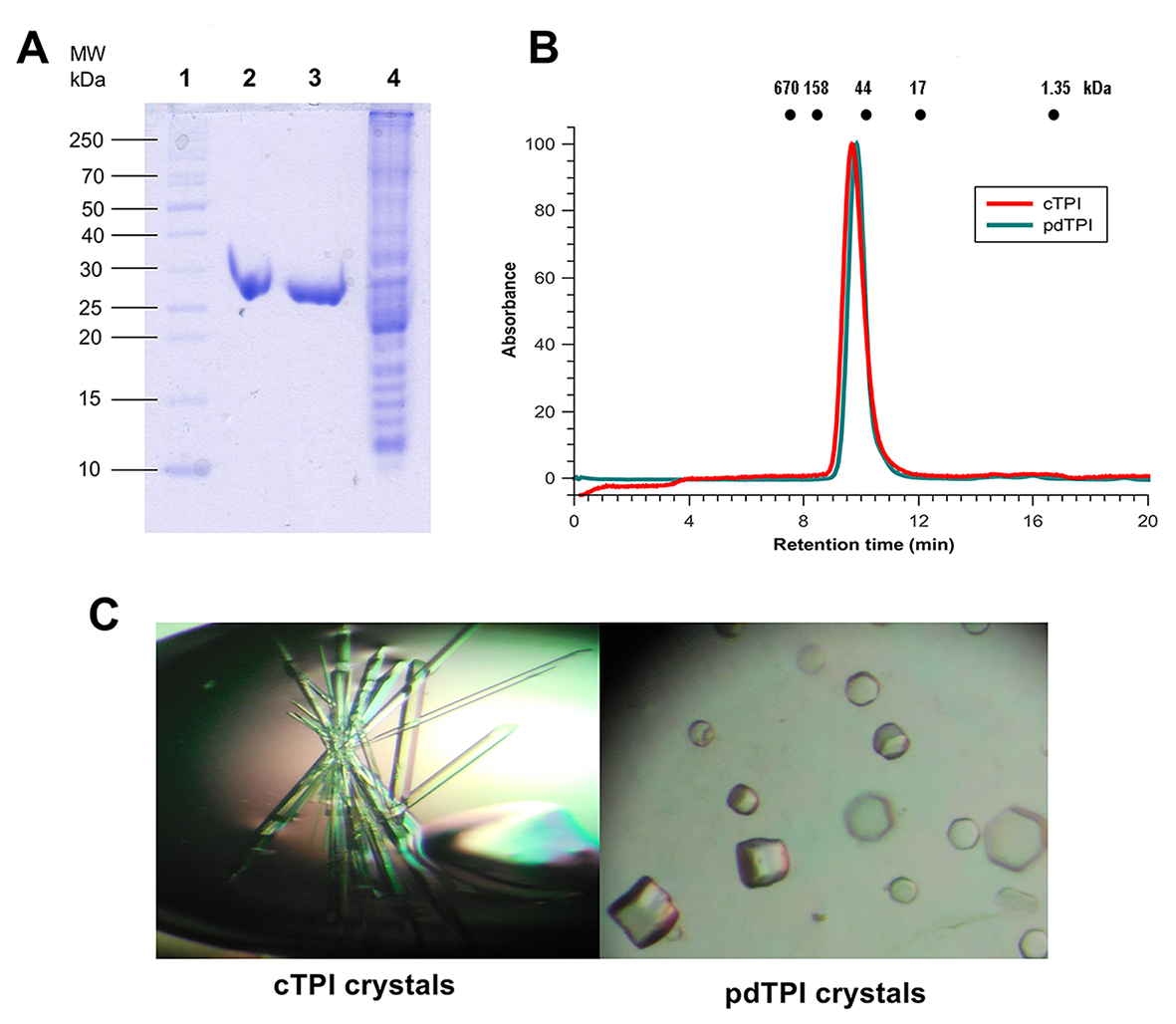

Supplement: Figure S2 — Purification, oligomeric state, and crystals from AtTPIs. (A) Eluate of cTPI (lane 2), pdTPI (lane 3), and sp-pdTPI (lane 4) after IMAC purification. Lane 1 indicates the MW ladder. (B) Chromatogram showing the elution profile of both AtTPIs. (C) Crystals obtained from cTPI and pdTPI used for X-ray diffraction and further resolution of both structures. [file Image2.TIFF]

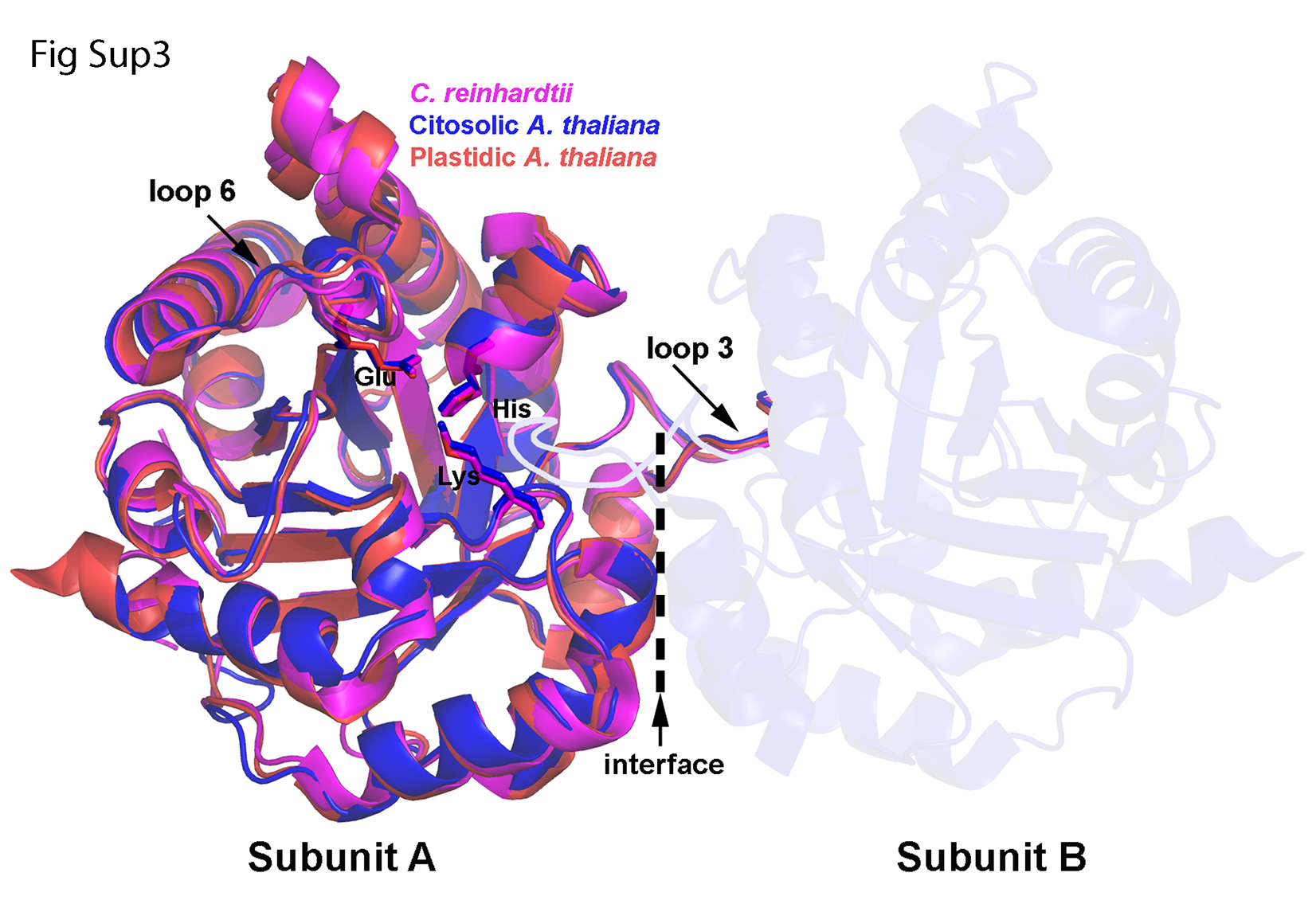

Supplement: Figure S3 — Structural comparison between AtTPIs and CrTP1. Superposition of the three crystal structures from photosynthetic organisms showing their active site and dimer interface. The catalytic residues Lys, His, and Glu are in a stick representation. Molecule B is presented to highlight the formation of the dimeric species and the interface. [file Image3.TIFF]

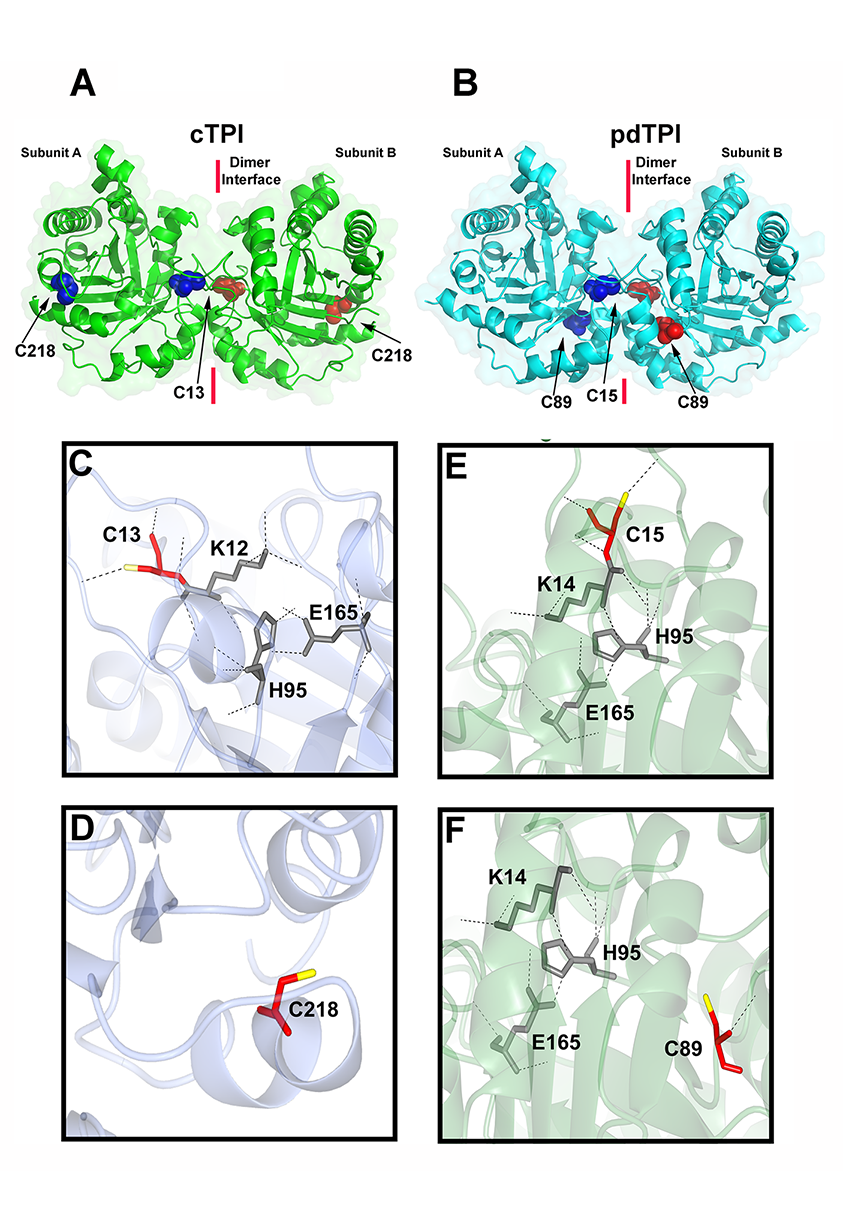

Supplement: Figure S4 — Crystal structures of AtTPIs. (A,B) Surface representation of the crystal structures of cTPI and pdTPI (green and cyan, respectively) showing the localization of their solvent exposed cysteines. (C) Structural localization of residue C13 on the cTPI crystal structure at the dimer interface. (D) Structural localization of residue C218 on the cTPI crystal structure. This residue is located at a distant position from the active site or the dimer interface. (E) Structural localization of C15 on the pdTPI crystal structure. As in cTPI-C13, this residue is part of the dimer interface. (F) Structural localization of C89 residue on the pdTPI crystal structure. The sulfhydryl group of pdTPI-c89 points toward the hydrophobic core. [file Image4.TIFF]

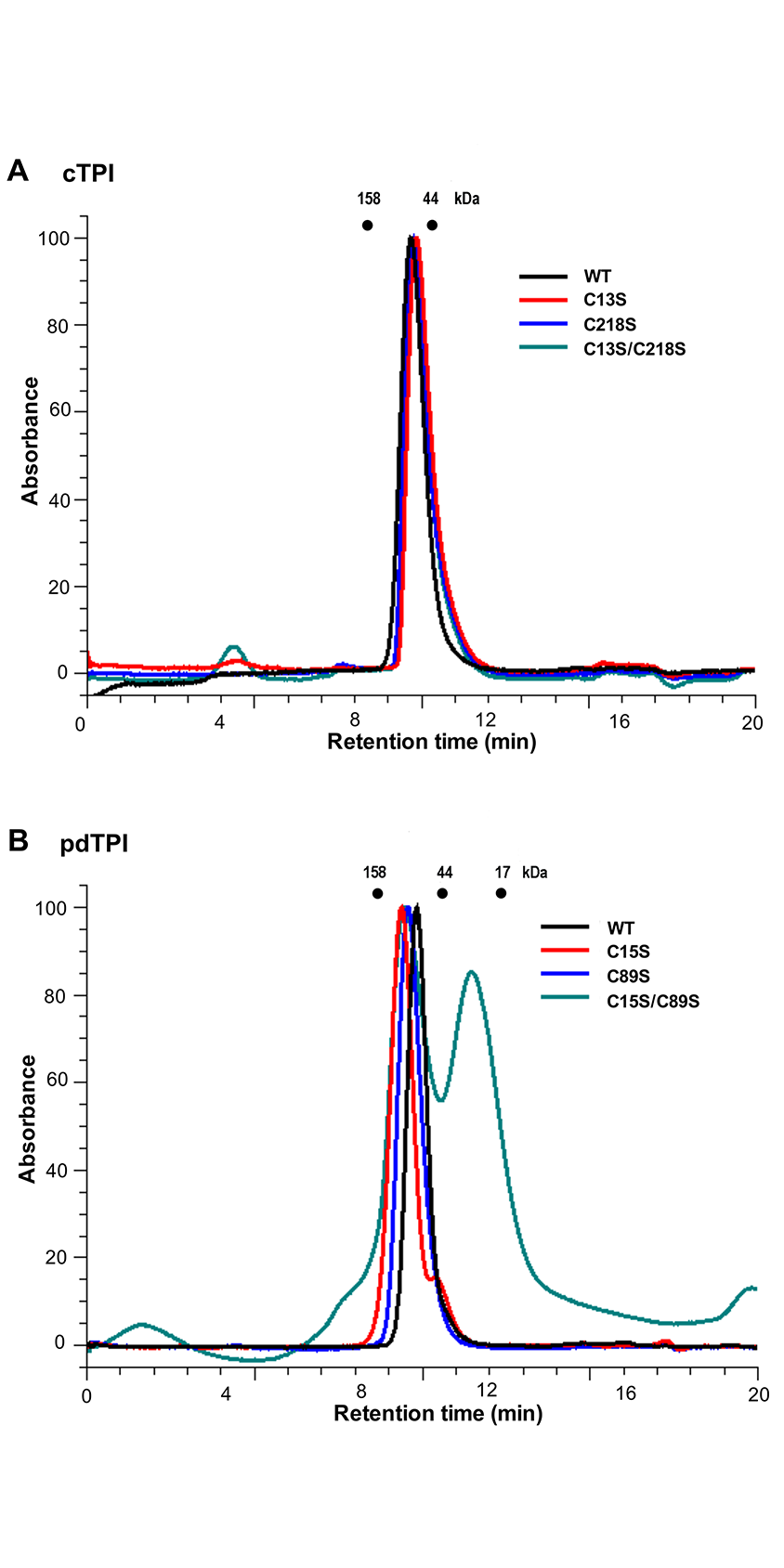

Supplement: Figure S5 — Single and double mutations alter the oligomeric state of AtTPIs. (A,B) Size-exclusion chromatography profiles showing the oligomeric state of single and double mutants of cTPI and pdTPI. The oligomeric state of cTPI is resistant to the effect of single and double mutants (A), whereas a double mutant of pdTPIC15S-C89S reduces the fraction of dimeric protein (B). [file Image5.TIFF]
