# Supplementary material for: Structural Basis for Redox Regulation of Cytoplasmic and Chloroplastic Triosephosphate Isomerases from Arabidopsis thaliana
Source: Front Plant Sci. 2016 Dec 6;7:1817. doi: 10.3389/fpls.2016.01817 (PMC5138414; doi:10.3389/fpls.2016.01817)
Supplement: Table S1 — Crystallographic data collection parameters and statistics. [file Table1.DOCX]

**Table 1S**. Crystallographic data collection parameters and statistics

| ***Identification*** | AtcTPI | AtpdTPI |
| --- | --- | --- |
| PDB code | 4OBT | 4OHQ |
|  |  |  |
| ***Data collection*** |  |  |
| Wavelength (Å) | 0.9786 | 0.9786 |
| Detector distance (mm) | 300 | 300 |
| Space group | P 2_1_ 2_1_ 2_1_ | P 6_5_ 2 2 |
| Resolution range (Å) | 38.97 - 1.6 (1.657 - 1.6) | 28.89 - 2.15 (2.23 - 2.15) |
|  |  |  |
| ***Unit cell parameters*** |  |  |
| *a*, *b*, *c* (Å) | 77.94 84.59 89.72 | 100.92 100.92 221.82 |
| α, β, γ (deg.) | 90 90 90 | 90 90 120 |
| No. of measured reflections | 577685 | 516488 |
| No. of unique reflections | 78866 | 37207 |
| Completeness ( %) | 99.97 (100.00) | 99.88 (99.92) |
| Redundancy | 7.32 | 13.88 |
| Mean I/σI | 13.02 (3.75) | 17.00 (4.60) |
| *R*_merge_ ( %) | 4.7 | 4.9 |
|  |  |  |
| ***Refinement*** |  |  |
| Resolution range (Å) | 38.97 – 1.60 (1.64 – 1.60) | 28.89 – 2.15 (2.20 - 2.15) |
| *R*_work_ / *R*_free_ ( %) | 16.62 / 18.89 | 17.00 / 21.17 |
| Reflections used (work/free) | 78784 /2000 | 37106 / 1998 |
| Number of molecules per asymmetric unit | 2 | 2 |
| Non-hydrogen atoms | 4088 | 3758 |
| Water molecules | 372 | 131 |
| Average B-factor (Å^2^) | 23.40 | 43.40 |
| Protein | 22.80 | 43.50 |
| Solvent | 29.60 | 39.90 |
|  |  |  |
| ***RMSD from ideal geometry*** |  |  |
| Bond lengths (Å) | 0.009 | 0.010 |
| Bond angles (deg.) | 1.14 | 1.15 |
|  |  |  |
| ***Ramachandran statistics*** |  |  |
| Favored ( %) | 98.4 | 97 |
| Outliers ( %) | 0 | 0.2 |
|  |  |  |
|  |  |  |

^a^Values in parentheses are for highest-resolution shell.

*R*_free_ is calculated from the randomly selected 5% (4WJE, 4OJS) set of reflections not included in the calculation of the *R*_work_ value.
